# Parallel ClickSeq and Nanopore sequencing elucidates the rapid evolution of defective-interfering RNAs in Flock House virus

**DOI:** 10.1371/journal.ppat.1006365

**Published:** 2017-05-05

**Authors:** Elizabeth Jaworski, Andrew Routh

**Affiliations:** 1 Department of Biochemistry and Molecular Biology, The University of Texas Medical Branch, Galveston, TX, United States of America; 2 Sealy Center for Structural Biology and Molecular Biophysics, University of Texas Medical Branch, Galveston, Texas, United States of America; University of Michigan, UNITED STATES

## Abstract

Defective-Interfering RNAs (DI-RNAs) have long been known to play an important role in virus replication and transmission. DI-RNAs emerge during virus passaging in both cell-culture and their hosts as a result of non-homologous RNA recombination. However, the principles of DI-RNA emergence and their subsequent evolution have remained elusive. Using a combination of long- and short-read Next-Generation Sequencing, we have characterized the formation of DI-RNAs during serial passaging of Flock House virus (FHV) in cell-culture over a period of 30 days in order to elucidate the pathways and potential mechanisms of DI-RNA emergence and evolution. For short-read RNAseq, we employed ‘ClickSeq’ due to its ability to sensitively and confidently detect RNA recombination events with nucleotide resolution. In parallel, we used the Oxford Nanopore Technologies’s (ONT) MinION to resolve full-length defective and wild-type viral genomes. Together, these accurately resolve both rare and common RNA recombination events, determine the correlation between recombination events, and quantifies the relative abundance of different DI-RNAs throughout passaging. We observe the formation of a diverse pool of defective RNAs at each stage of viral passaging. However, many of these ‘intermediate’ species, while present in early stages of passaging, do not accumulate. After approximately 9 days of passaging we observe the rapid accumulation of DI-RNAs with a correlated reduction in specific infectivity and with the Nanopore data find that DI-RNAs are characterized by multiple RNA recombination events. This suggests that intermediate DI-RNA species are not competitive and that multiple recombination events interact epistatically to confer ‘mature’ DI-RNAs with their selective advantage allowing for their rapid accumulation. Alternatively, it is possible that mature DI-RNA species are generated in a single event involving multiple RNA rearrangements. These insights have important consequences for our understanding of the mechanisms, determinants and limitations in the emergence and evolution of DI-RNAs.

## Author summary

Defective RNAs are versions of a viral genome that arise naturally during viral infections but have been truncated or rearranged by non-homologous recombination. While not encoding for functional viruses, they can be amplified and co-passaged with the wild-type virus, effectively parasitizing the normal viral machinery. Some defective RNAs can replicate so successfully so as to subdue the replication of the wild-type virus, forming a ‘*Defective-Interfering RNA’* (DI-RNA). As a result, DI-RNAs may promote the establishment of chronic viral infections, may prolong the period during which the host is infectious, and may even be exploited as antiviral therapies or vaccines. Therefore, understanding the reasons and mechanisms of how DI-RNAs are formed and subsequently evolve is important. Here, we sought to characterize these processes by passaging Flock House virus (a highly tractable and well-characterized model RNA virus) in cell-culture for approximately one month and sequencing the viral genomes every three days using a combination of ‘ClickSeq’ (to resolve recombination events with nucleotide resolution) and the Oxford Nanopore Technologies MinION (to characterize full-length and defective genomes). This provides a highly detailed characterization of the pathways of DI-RNA emergence and their progression to dominance over the wild-type viral genome.

## Introduction

RNA viruses are extremely diverse and rapidly evolving. Their RNA-dependent RNA polymerases (RdRps) readily generate single-nucleotide variants whilst lacking proof-reading capabilities[1]. RdRps are also highly prone to RNA recombination[2]; either through template-switching[3] or through non-replicative end-joining[4]. RNA recombination has been demonstrated to be responsible for the emergence of new strains or species of viruses such as rhinoviruses[5] and dengue virus[6], and the formation of vaccine-derived poliovirus[7]. Non-homologous RNA recombination is also responsible for the generation of defective RNAs[8, 9]. These are versions of the parental viral genome that can arise naturally during the course of viral passaging but have been truncated and rearranged by RNA recombination. While not encoding for functional viruses themselves, they can be amplified and co-passaged with the help of the wild-type ‘helper’ virus that provides the necessary machinery for replication, encapsidation and transmission. A defective RNA that accumulates to such an extent as to compete with or otherwise attenuate the replication of the parental virus is known as a Defective-Interfering RNA (DI-RNA)[10].

DI-RNAs can attenuate the viral infection via a variety of proposed mechanisms such as the saturation of the viral replicative machinery, sequestration of essential cellular cofactors, and/or induction of innate immune responses[10–14]. DI-RNAs have been well characterized for a number of RNA viruses as they provide valuable tools to molecular virologists by revealing conserved regions and functional domains in the RNA genome such as binding sites for viral or host factors. Moreover, characterizing recombination loci reveal the mechanisms of recombination, impacting our understanding of viral evolution [8, 15, 16].

Until recently [17], due to difficulties in capturing and characterizing DI-RNAs *in vivo*, DI-RNAs were considered to be a curious epiphenomenon of cell-culturing practices. As a result, our appreciation of the diversity of DI-RNAs and the range of situations in which they could play a role was greatly limited. Increasingly, due to the use and sensitivity of Next-Generation Sequencing (NGS) technologies, DI-RNAs have been observed in a multitude of viral systems under laboratory conditions (e.g. SARS coronavirus[18], HIV[19]), in clinical settings (e.g. measles[20], dengue[21] and chronic hepatitis C[22]) and in metagenomic or ‘wild’ samples (e.g. West Nile virus[23], influenza virus[24]). Despite this burgeoning range of hosts for DI-RNAs, limitations in NGS technologies including high artifactual recombination rates, short reads and a limited range of bioinformatics tools tailored to viral RNA recombination discovery has hindered our ability to detect and characterize DI-RNAs in complex or clinical samples.

Flock House virus (FHV) is a positive-sense single-stranded RNA (+ssRNA) virus originally isolated from grass grubs in New Zealand[25] and is perhaps the best-studied *Alphanodavirus* from the *Nodaviridae* family. FHV infects *Drosophila* flies and cells in culture as well as medically important genera of insects including mosquitos, (*Anopheles gambiae)*, the tsetse fly (*Glossina morsitans morsitans Westwood*), and the Chagas vector (*Rhodnius prolixus Stal)*[26]. Infection of these organisms by FHV has been demonstrated to have similar characteristics in terms of viral titer, virus dissemination and mortality as has been shown for fruit fly infections. FHV provides an excellent model system to study +ssRNA virus evolution by virtue of having one of the smallest known eukaryotic virus genomes[27]. Moreover, the viral life-cycle and details of the molecular biology of virus particle assembly, cell entry and particle disassembly are highly-characterized. FHV contains two genomic RNAs. RNA1 (3107 nts) encodes the viral RdRp and RNA2 (1400 nts) encodes the viral capsid protein. RNA1 also expresses a small sub-genomic RNA, called RNA3, that encodes the B2 protein responsible for inhibition of the anti-viral RNAi machinery[28]. FHV has been demonstrated to form DI-RNAs in multiple independent studies spanning three decades both in cell-culture[8, 26, 29–32] and in *Drosophila melanogaster*[33]. Many of these studies characterized individual DI-RNA genomes through sub-cloning and Sanger sequencing. Intriguingly, many of these DI-RNAs are highly similar. This indicates that either the DI-RNAs have emerged due to a common mechanism of formation or the presence of a common selectivity filter, or both. Our recent NGS studies of RNA recombination in FHV revealed a diverse array of RNA recombination events, suggesting that the genomic landscape of DI-RNAs is highly dynamic and likely contributes significantly to the diversity of viral genomes that form the viral quasi-species[34]. Despite these findings, studies to-date present only a single snap-shot of the DI-RNA genome landscape and do not capture the pathways of their emergence and evolution nor characterize any intermediate DI-RNA species that might arise during these processes.

In order to resolve the potential mechanisms of DI-RNA emergence and elucidate the evolutionary pathways that lead to the formation of ‘mature’ DI-RNAs, we performed high-titer serial passaging of FHV in cell culture and characterized the encapsidated RNA using RNAseq. We used Illumina HiSeq sequencing of ClickSeq generated libraries to provide a high-resolution and high-confidence quantification of individual recombination events. We combined this information with long-read Oxford Nanopore Technologies’s (ONT) MinION sequencing to resolve the topology of full-length and defective RNA genomes. By combining these data, we aimed to determine the correlation of recombination events within single RNA virus genomes, characterize the distribution of defective RNA genomes, and determine the exact make-up of DI-RNAs during serial passaging of FHV in cell culture.

We recently developed the ‘ClickSeq’ method for RNAseq that uses copper-catalyzed alkyne-azide cycloaddition (CuAAC), a click-chemistry reaction, for RNAseq library synthesis[35]. ClickSeq provides a robust platform on which to study RNA recombination in RNA viruses. Artifactual recombination is a common contaminant in NGS library generation and can easily obscure rare or non-canonical recombinant species. ClickSeq does not require template fragmentation and replaces enzymatic ligation steps commonly required in NGS library generation with click-chemistry. ClickSeq works by introducing small amounts of azido-nucleotides (AzNTPs) into RT-PCR reactions to generate azido-terminated cDNA transcripts. These cDNA fragments are subsequently mixed with alkyne-labelled DNA adaptors. The addition of a copper catalyst results in the ‘click-ligation’ of the two chemically-functionalized DNA substrates to produce an unnatural triazole-linked single-stranded DNA molecule[36]. ClickSeq prevents template switching during RT-PCR as well as non-specific ligation of RNA fragments. We demonstrated that ClickSeq reduces artifactual recombination to fewer than 3 events per million mapped reads[35]. As a result, ClickSeq provides a superior method for the detection of DI-RNAs and RNA recombination events.

The Oxford Nanopore Technologies’s (ONT) MinION is a small handheld sequencing device[37] poised to revolutionize the next-generation sequencing field by providing real-time, high-throughput and long-range (over 882 kbp[38]) sequences of DNA samples with minimal sample prep. ONT nanopore sequencing has been used to rapidly characterize virus genomes from metagenomic samples[39], in the midst of Ebola virus outbreaks[40], and in targeted studies aimed at characterizing sequence variations within influenza virus samples[41]. Highly parallel direct RNA sequencing using Nanopore technology was also recently reported[42]. Due to the higher error-rate[43] of the nanopore sequencing technology compared to other RNAseq platforms, the exact identity of recombination events within single-molecule genomes may be inaccurate. However, long-read nanopore reads provide the distinct advantage of being able to sequence full-length cDNA copies of RNA virus genomes and thus can resolve multiple recombination events within a single RNA virus genome.

This study provides a comprehensive analysis of the steps and pathways governing DI-RNA emergence and evolution starting from a plasmid-driven inoculum through to a highly-passaged sample. By combining short-read and long-read sequencing technologies, we determine both the exact identity of RNA recombination sites and their correlation within the viral quasispecies. We find little evidence for the accumulation of intermediate defective RNA species that contain either only one, or smaller, deletions during the course of passaging. Rather, fully formed ‘mature’ DI-RNAs that are characterized by two to three deletions between a limited number of positions in each of the FHV genomic RNAs appear after approximately 9 days of viral passaging and accumulate rapidly. The accumulation of DI-RNAs corresponds with a reduction in the specific infectivity of the viral samples in each passage. This implies that partially formed DI-RNA species are not competitive and cannot accumulate in the manner that mature DI-RNA species do, perhaps due to the epistatic interaction of multiple recombination events. Alternatively, the formation of mature DI-RNAs may occur in a single step involving multiple simultaneous genome rearrangements.

## Methods

### Cell culture and virus passaging

*D*. *melanogaster* (S2) cells were grown at 28°C in Schneider’s *Drosophila* Media supplemented with 10% fetal bovine serum and 1X Penicillin-Streptomycin using standard laboratory procedures. To generate the initial Flock House virus inoculum, S2 cells were plated at 50–70% confluency in a six well plate and were transfected with 2.5μg of pMT plasmid containing FHV RNA1 (NC_004146) and 2.5μg of pMT plasmid containing FHV RNA2 (NC_004144) using Lipofectamine 3000 Transfection Reagent as per the manufacturer’s protocol. Plasmid transcription was induced 24 hours post transfection with the addition of 50mM CuSO_4_. Virus was then allowed to propagate for 3 days post induction. For successive passages (Passages 1–9), S2 cells were grown in T-25 flasks to 70–90% confluency (~1 x 10^7^ cells), then infected with 1mL of viral inoculum from the previous passage. Virus was grown for 3 days, then fractions were harvested for viral purification or inoculation of the next passage.

### Virus isolation and purification

To purify virus from each consecutive serial passage, cells and supernatant were subjected to a freeze-thaw cycle in the presence of 1% Triton X-100 to release viral particles from infected cells. Virus particles were then purified on a 30% sucrose cushion by spinning the cell lysate at 40,000 RPM for 2.5 hours. The viral pellet was resuspended in 10mM Tris (pH 7.4). Virus was further purified by applying resuspended virus atop a 10–40% sucrose gradient and spun at 40,000 RPM for 1.5 hours. The viral band was collected and subsequently treated with 1 Unit DNase and 1 Unit RNase and incubated at room temperature for at least one hour to remove any cellular nucleic acids not protected by the viral capsid. The virus sample was concentrated on a 100,000 NMWL centrifugal filter column and washed with at least 2 volumes of 10mM Tris pH 7.4. Finally, encapsidated viral RNA was extracted using a QIAGEN RNeasy Mini Kit as per the manufacturer’s protocol.

### Short-read Illumina sequencing of viral RNA

Next generation sequencing (NGS) libraries were generated using 100ng of RNA using the ‘ClickSeq’ protocol as previously described by Routh et al. [35, 44, 45]. Briefly, cDNA is synthesized through RT-PCR initiated from semi-random (6N) primers containing a partial Illumina p7 adapter (GTGACTGGAGTTCAGACGTGTGCTCTTCCGATCTNNNNNN) and stochastically terminated by the addition of azido-NTPs (AzNTP) at a ratio of 1:35 AzNTP:dNTPs. Subsequently, the p5 Click-Adapter (5’-Hexynyl-NNNNAGATCGGAAGAGCGTCGTGTAGGGAAAGAGTGTAGATCTCGGTGGTCGCCGTATCATT, IDT) was click-ligated onto the azido-terminated cDNA fragment using copper-catalysed azide-alkyne cycloaddition (CuAAC) in the presence of TBTA ligand (Lumiprobe) and Vitamin C catalyst in 55% DMSO. After purifying the click-linked cDNA with a Zymo DNA clean column, 18 cycles of OneTaq (NEB) PCR amplification adds the remainder of the p7 adapter along with the desired TruSeq index sequence. PCR product was cleaned again with a Zymo DNA clean column to remove excess primers and then ran on a 1–2% precast agarose e-gel (Invitrogen, E-Gel Electrophoresis System). cDNA libraries between 400 to 700bp were excised corresponding to insert sizes of 250-550bp and cleaned using the Zymo Research Gel DNA Recovery Kit. Final cDNA libraries were quantified using a QuBit fluorimeter (Life Tech) and loaded on a HiSeq 1500 single read rapid run flowcell for 1x150 reads and 7 nucleotides of the index. FHV libraries used for the triplicate study shown in **S2 Fig** were sequenced on a MiSeq platform with v3 chemistry for 600 cycles (2x300). Reads were trimmed to 150nts prior to analysis to emulate the libraries sequenced on the HiSeq.

### Illumina data analysis and processing

Raw reads were processed by first removing the Illumina TruSeq adaptor using *Cutadapt* [46] with default parameters. Next, the first 6 nucleotides (corresponding to the random nucleotides and triazole-linkage included in the Click-Adaptor) were trimmed and any reads that contained nucleotides with a PHRED score <20 were removed using the *FASTX toolkit* (http://hannonlab.cshl.edu/fastx_toolkit/). The remaining reads were aligned end-to-end with *Bowtie* (v1.0.1) [47] (command line parameters: -v 3 –-best) first to the FHV genome (NC_004414 and NC_004146) and next to host *D*. *melanogaster* genome (fb5_22). The remaining unmapped reads were processed to identify recombination events using the python script ‘*ViReMa*’ (Viral Recombination Mapper)[32] (command line parameters:--N 1 --X 5 --Seed 25 --Host_Seed 30 --Defuzz 0 --MicroInDel 5). The frequency of a specific recombination event is approximated by dividing the number of reads mapping to this recombination (*N*) by *N* plus the average of the number of reads mapping to the wild-type genome at each of the recombination coordinates.

### Long-read Oxford Nanopore technologies’s MinION sequencing

The Oxford Nanopore Technologies’s (ONT) MinION and flowcells were acquired as part of the ONT early-access program. To prepare sequencing libraries for the MinION, 50ng of RNA was reverse transcribed using RNA specific primers that were complimentary to the 3’ end of the respective genome (RNA1_RP: ACCTCTGCCCTTTCGGGCTA or RNA2_RP: ACCTTAGTCTGTTGACTTAA). cDNA was then amplified using the standard Phusion (NEB) PCR protocol using genome specific primers (RNA1_FP: GTTTTCGAAACAAATAAAAC or RNA2_FP: GTAAACAATTCCAAGTTCCA) for 19 cycles. Excess primers were removed from the PCR product using AMPure XP beads (Beckman Coulter) at a ratio of 1:1 AMPure bead:PCR product. Samples were then barcoded and prepared following the manufacture’s protocol (R9 Native Barcoding Kit I and Nanopore Sequencing Kit) with adjustments to tailor input cDNA quantities. A target of 1ug of fragmented DNA at approximately 8’000 nts is considered optimal for library generation using this kit. The input amounts for RNA1 (3107 bp) and RNA2 (1400 bp) were thus adjusted to 192ng and 88ng respectively and combined in 46uL water to maintain optimal DNA end molarity. After ligation of barcodes, equal amount of each DNA library (9 samples in total) were pooled and loaded onto a MinION MkIB device equipped with an R9 flow cell. The MinKNOW control software was used to select a 48-hour sequencing protocol and was allowed to proceed for at least 36 hours, until high-quality data accumulation ceased. Raw data was uploaded automatically by Metrichor software for cloud-based base-calling using default settings and quality filtering for 2-dimensional reads. Reads were extracted from HDF5 format files (fast5) using *poretools*[48].

### ONT nanopore data processing and alignment

Full-length ONT reads were mapped to the Flock House virus genome using the pacbio wrapper from the *BBMAP* v36 suite (command line parameters: fastareadlen = 6000 vslow = t maxindel = 3100 minid = 0.5 local = f ignorebadquality = t usequality = f). Alignment SAM files were visualized using the Tablet sequence viewer [49]. SAM files were filtered to ensure that MinION reads mapped from the first 25 nts to the final 25 nts of the reference genome (accounting for deletions and insertions), due to the presence of truncated nanopore reads and mis-priming during the cDNA PCR amplification steps. Errors including substitutions, insertions and deletions were counted using the *samtools[50] mpileup* command and error rates at each position were calculated by dividing this value by the read depth at this position (**S5 Fig**). Insertion and deletion events longer that 25 nts were extracted using the CIGAR string of the SAM files using simple in-house scripts. For recombination sites containing ‘fuzz’, where nucleotides surrounding the putative recombination events are the same for both the acceptor and donor sites, the recombination event was reported as occurring in the middle of the ‘fuzzy’ region, or at the 5’ side of the middle two nucleotides in the orientation of the reference if the fuzzy site contained an even number of residues. This is the same methodology as employed in the *ViReMa* script[32] used to map recombination event in the ClickSeq data. Insertion events and soft-pads longer than 100 nts were extracted and their nucleotide sequence was analyzed using an online BLASTn search to determine their identity.

### Annotation of full-length defective RNAs and recombination events

To annotate the defective genomes detected by MinION nanopore sequencing or recombination events detected by ClickSeq, we use underscores ‘_’ to denote continued mapping, and carets ‘^’ to denote a recombination events. For example, “*1_317^1091_1242^2301_3107*” indicates an authentic mapping from nt 1 to 317, then a deletion event removing nts 318 through 1090, then another authentic mapping from 1091 to 1242, followed by another deletion removing nts 1243 through 2300, and finally an authentic mapping from nt 2301 to 3107.

### Shannon entropy index

The Shannon Entropy Index is given by: $H(X)=-\sum_{i=0}^{N-1}\;p_{i}\ln\;p_{i}$ For the ClickSeq data, each recombination event is treated as independent with its probability determined by dividing the number of reads mapping to the present recombination event divided by the average coverage over the whole viral RNA. For the MinION data, each individual read mapping is treated as an individual event with the frequency determined by dividing the number of identically mapping reads divided by total mapped reads.

### Effective MOI and specific infectivity

Tissue culture infective dose 50 (TCID50) analyses of the supernatants from each passage were performed using standard protocols [51]. For purified particles of each passage TCID50 was calculated with slight modifications. Specifically, 1 x 10^5^ cells (S2) per well were plated in 96 well format. Virus samples were quantified by measuring the OD_260nm_. An OD of 4.15 corresponds to 1mg virus [52], which in turn corresponds to 6.4 x 10^13^ virus particles assuming a virion mass of 9.4MDa [53]. Purified virus samples were diluted to a starting concentration of 47ng/μL, which corresponds to 3 x 10^10^ virus particles per 10μL. These quantities were chosen as particle-to-PFU ratios for rescued FHV has previously been reported to be 300–400 particles [26, 52]. Therefore 3 x 10^10^ particles per 10^5^ cells corresponds to approximately 1000 PFUs per cell. Eight serial 10-fold dilutions were subsequently made and added to each column of the 96-well plate (8 replicate wells per dilution), as per standard TCID50 protocols. Virus was allowed to grow for 4 days after which the number of positive wells exhibiting cytopathic effect (CPE) were counted. The TCID50 values and effective MOI were calculated using the Reed and Muench Calculator [51]. We further counted the total number of cells that were present in each well after infection using a Guava easyCyte HT (Millipore) flow cytometer to provide us with quantifiable amount of cell death. 50μL from each well was diluted in 150μL PBS and injected using manufactures’ protocols. The InCyt v3.1 software was used to collect data with the following parameters: collection time: 30sec; flow rate: 0.59μL/sec; FSC: 16; SSC: 25; threshold: 0. The region used to determine live cells was based on scattering features of the negative controls (wells with no viral infection) (**S7 Fig**).

### Accession numbers

All raw Illumina data and demultiplexed MinION nanopore data passing quality filters (comprising 2D, template and complement strands) associated with this manuscript are available on the SRA NCBI archive with study number SRP094723 and BioProject number PRJNA352872.

## Results

### Serial passaging of Flock House virus

**Fig 1:**
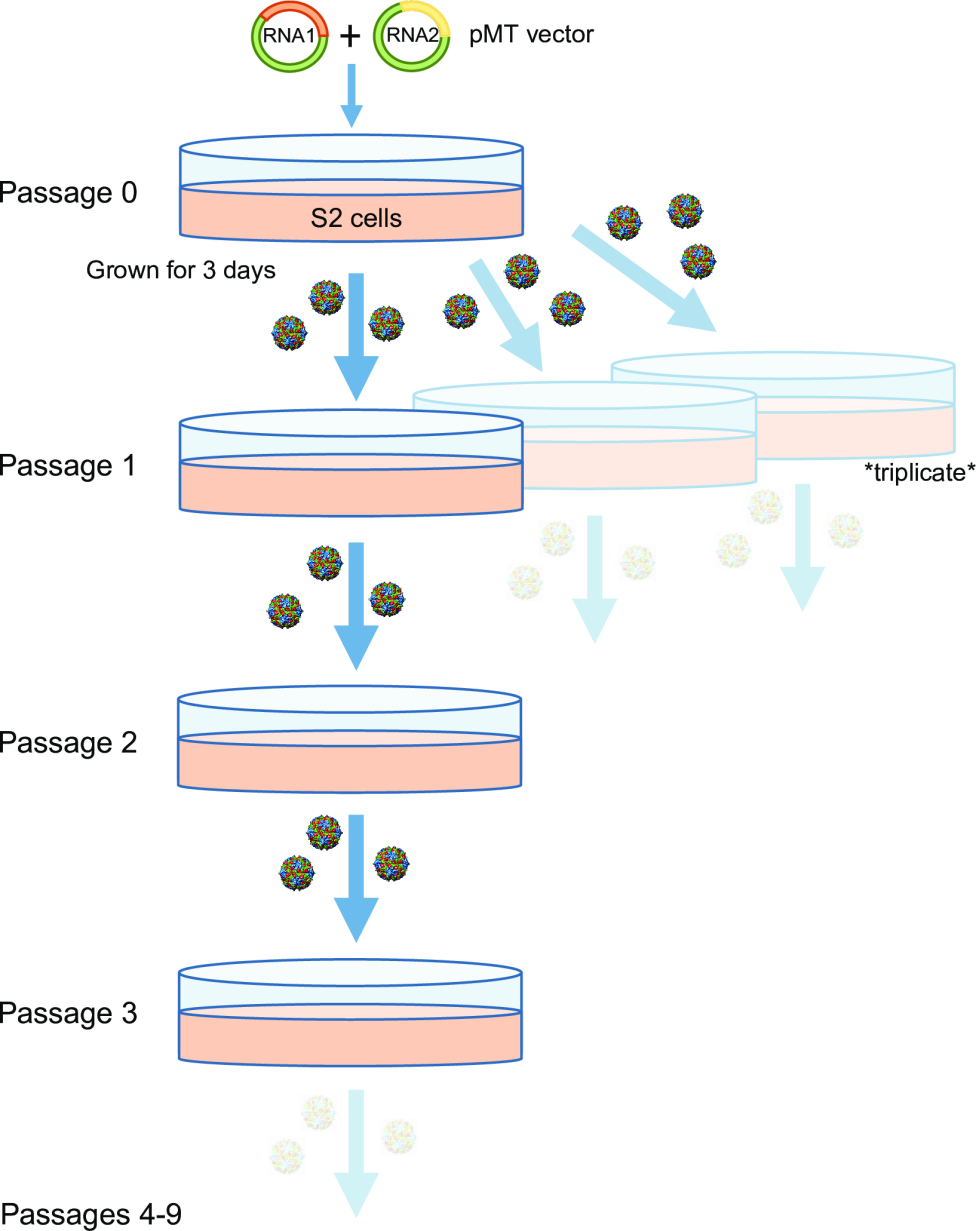
Serial passaging of Flock House virus in *Drosophila melanogaster*. S2 cells were transfected with pMT vectors containing either FHV RNA1 or RNA2 and induced to generate genetically homogeneous viral particles. Virus was allowed to propagate for three days after which cells and the supernatant were collected. A fraction (1:10) was used to further infect a new population of S2 cells in a series of passages. The remaining fraction was collected for analysis. In total, three replicates of nine passages were collected.

*D*. *melanogaster* (S2) cells in culture were transfected with cDNA plasmids containing each of the Flock House virus genomic RNAs followed by a hepatitis D virus (HDV) ribozyme sequence. After induction, the HDV ribozyme regenerates the authentic 3’ end of the positive sense viral RNA, which is thus successfully recognized by the FHV RdRp allowing the initiation of viral replication[54]. We choose to initiate replication with this method to ensure that the starting viral population would be homogeneous containing only the full-length RNAs derived from the plasmid cDNA. After transfection, the viral inoculum was allowed to amplify for 3 days (Passage number = P0), after which most cells exhibited cytopathic effect. Subsequently, the supernatant from infected cells was collected and a 1mL fraction (10% of the total volume) was used to infect 10mL of fresh S2 cells in triplicate (Replicates R1, R2, and R3). Again, after three days, 1mL of the supernatant was harvested and used to infect fresh S2 cells in series for a total of nine 3-day passages (Passage numbers = P1 –P9). Therefore, one single inoculum was used to generate three distinct lineages as shown in **Fig 1**. For each passage and replicate, including the original inoculum, viral particles were purified over a sucrose cushion and non-encapsidated genetic material was degraded to ensure that the genetic material subsequently analyzed was packaged within the viral capsid. RNA was extracted from the purified viral particles using standard silica-based spin columns.

### Characterization of FHV genomic RNA with short-read ClickSeq sequencing

ClickSeq libraries[35] were synthesized from the purified viral genomic RNA and sequenced on an Illumina HiSeq 1500 for 1x150 single-end reads. The inoculum sample was sequenced on a separate flowcell to all other samples to prevent any cross-contamination from incorrect demultiplexing. We obtained 1.2–30.6 million reads after trimming and quality filtering for each passaged sample, and 41.6 million reads for the original inoculum (**Table 1**and **S1 Table)**. 1 million reads corresponds to an average coverage of greater than 33’000X across the FHV genome. Reads were aligned to the FHV genome and the host genome (*D*. *melanogaster*, fb5_22) using *Bowtie* end-to-end mapping [47]. As expected, the majority of the reads aligned to FHV RNA1 (3107 nts) and FHV RNA2 (1400 nts) in a ratio reflecting the longer length of RNA1. As we have observed previously [34, 55], 0.3–8.4% of reads correspond to host RNAs that are encapsidated within the viral particles including mRNAs, ribosomal RNAs and retrotransposons. Interestingly, the amount of encapsidated host RNA increases modestly through later passages **(S1 Fig).**

**Table 1:** Number of ClickSeq reads gathered from each passage of replicate 2.

*Replicate 2*	Passage 0	Passage 1	Passage 2	Passage 3	Passage 4	Passage 5	Passage 6	Passage 7	Passage 8	Passage 9
**Total Reads**	41,578,802	11,303,746	30,630,462	15,720,784	9,438,862	9,526,140	7,118,697	4,316,239	9,249,953	8,108,519
**FHV Genome**	39,595,900	11,121,111	29,693,227	15,243,341	9,229,809	8,996,228	6,293,323	3,953,100	8,162,144	7,088,432
**Host (*D*. *mel*)**	1,057,037	100,716	596,462	171,518	29,353	81,172	286,605	71,238	270,156	404,742
**FHV Recombs**	161,205	9259	46,962	192,737	126,061	377,703	409,316	246,959	679,389	437,566
RNA1-RNA1	2,902	5103	25,052	24,809	46,225	241,048	333,125	204,609	566,092	376,893
RNA2-RNA2	81,765	3845	20,919	167,464	79,550	136,141	75,424	42,076	111,895	59,650
Inter-RNA	76,538	311	991	464	286	514	767	274	1402	1023
Other	141,332	7358	33,488	16,642	8934	18,569	33,152	16,553	46,621	55,529
**Unmapped**	139,057	3779	9879	3026	2952	3569	4084	1631	2690	3549

Quantity of reads generated from an Illumina HiSeq run for each passage are tabulated. Reads were mapped to either FHV or the host using *Bowtie*. Remaining reads were then processed using *ViReMa* which identifies recombination events. ‘Inter-RNA’ indicates recombination events between RNA1 to RNA2 or vice-versa. ‘Other’ indicates reads that contain unknown/ambiguous recombination events and unmapped read segments.

**Fig 2:**
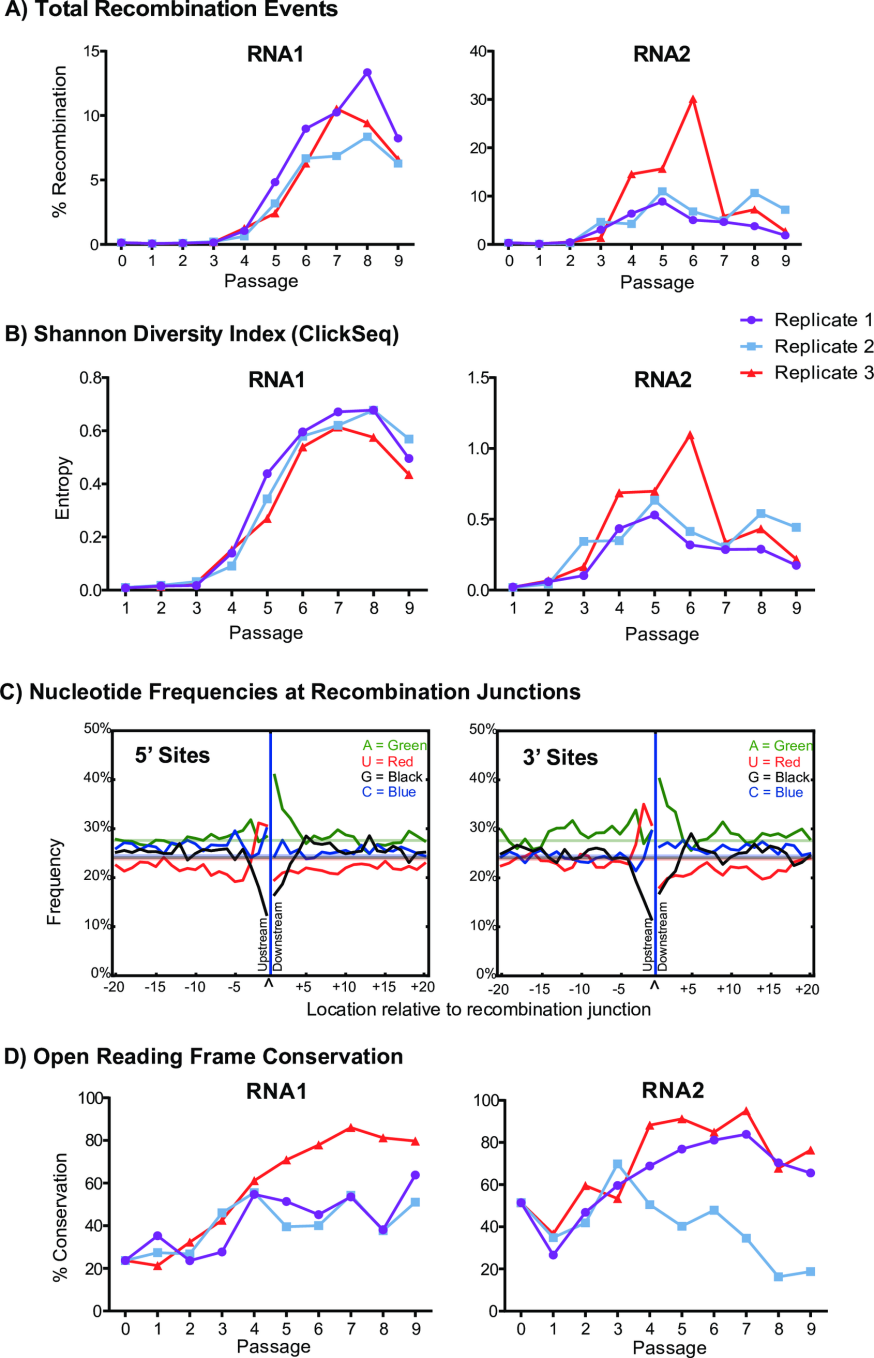
RNA recombination is characterized using RNAseq during serial passaging of FHV in cell-culture. **(A)** Frequency of recombination events and **(B)** Shannon Diversity Index for all passages and replicates. Percent recombination is calculated from total number of FHV recombination events per total number of reads mapped to FHV. **(C)** The frequency of each nucleotide found both upstream and downstream of the 5’ and 3’ sites of RNA recombination events are plotted. Only recombination events with fewer than 10 reads were included in this analysis to avoid over-sampling of highly replicated DI-RNAs. The composition (and therefore expected frequency) of nucleotides in the FHV genome is given by the colored horizontal lines in each plot. **(D)** A recombination event can retain a putative open reading frame by deleting 3n nucleotides. The frequency of conservation of the reading frame is calculated from the ratio of the number of recombination events that delete 3n nucleotides to the total number of recombination events mapped to that RNA.

Subsequently, we further characterized the unmapped reads with the Python script *ViReMa* (“Virus Recombination Mapper”) [32]. *ViReMa* is a computational pipeline optimized for mapping virus recombination junctions in NGS data with nucleotide resolution by dynamically generating moving read segments. *ViReMa* is sensitive to many types of RNA recombination events. This includes micro-insertions and deletions (InDels comprising 5 or fewer nucleotides), duplications, deletions, inter-RNA recombination (denoting recombination between FHV RNA1 and RNA2) and virus-to-host recombination events, and reports both the identity and frequency of recombination events. Recombination events that cannot be unambiguously identified due to unmapped read segments, mismatches occurring near to putative recombination events, or reads containing fragments of sequencing adaptors are flagged as ‘*other’* **(Table 1)** [32]. In each genomic RNA we found hundreds of unique recombination events, reflecting a diverse and complex mutational landscape (**S1 Datafile**). Broadly, we see an increase in the total number of recombination events during serial passaging of FHV and a corresponding increase in the Shannon diversity index (**Fig 2A and 2B**).

Following these mapping procedures, few reads (0–1.2%, **Table 1**and **S1 Table**) remained uncharacterized. As in previous studies, these were found to be derived from incorrect demultiplexing of neighboring samples on the HiSeq flow-cell[56] or from contaminants in the RNAseq library generation[57]. Having accounted for almost all of the reads present in each dataset, we can be confident that we are capturing the full range of recombination events and/or other rearrangements present within each sample and thus are not missing important or significant events due to computational limitations.

To demonstrate the reproducibility of the ClickSeq approach and to assess the limit in terms of our ability to successfully detect rare recombination events, we generated three replicate ClickSeq libraries from the RNA sample P7R2, obtained 1x150bp reads and performed the same computational analyses as described above. We mapped 0.83M, 1.45M and 1.18M reads per library, giving an approximate coverage of ~42,000–139,000x coverage over FHV RNA1 and ~18,000–67,000x over FHV RNA2 (calculated from the average coverage over the conserved 5’ and 3’ ends). When comparing the frequency of unique recombination events in either RNA1 or RNA2 between any pair of the three replicates, we find excellent correlation (Pearson >0.99) even for very infrequent recombination events, as illustrated in the scatter plots in **S2 Fig**. Events that were found in two replicates but not a third, never exceeded more than 20 mapped reads for RNA1 and 8 reads for RNA2. If we take these values as a cut-offs, below which we begin to fail to detect events, then we can conservatively estimate that we are reproducibly sensitive to recombinant species that are present at approximately 0.048% (20/42,000) of RNA1 population and 0.044% (8/18,000) of the total RNA2 population when obtaining ~1M sequence reads.

### Recombination profiling reveals emergence and accumulation of DI-RNAs

In the inoculum (P0), less than 0.2% of the all the reads mapped to recombination events (**Table 1, Fig 2A**). Inspection of these events reveals that they are dispersed throughout each of the genomic RNAs. RNA1 recombination events are the least frequent, with only 22 unique events detected represented by 2920 reads and without an apparent bias toward any specific location. The three most common RNA1 events in the inoculum are 2325^1241, 1484^1944, and 2199^393 with 514, 229, and 177 mapped reads respectively (**S1 Datafile**). Read depth for the wild-type genome at these loci ranges from 400K to 1.9M reads, therefore these recombination events make-up at less than 0.1% of the total viral population. These are not the events that have been previously reported as forming FHV DI-RNAs, moreover none of these events are observed again in subsequent passages, perhaps due to approaching the sensitivity of discovery limit as described above. However, due to the low-rate of artifactual recombination of the ClickSeq approach (>3 events per millions reads [35]), we can be confident that these are not sequencing artifacts[35]. Therefore, these events likely represent non-viable or transient recombination events that arose due to stochastic non-homologous recombination.

For RNA2 in the inoculum, the three most frequently observed events were 738^1219, 738^1222 and 1024^1190, with 9849, 5237, and 3673 mapped reads respectively (**S1 Datafile**). Coverage in the wild-type RNA2 mapping in these regions ranges between 2.6M and 4.8M mapped reads, therefore these recombinant species make-up approximately 0.2% of the viral RNA2 population. The majority of other recombination events are found to delete a similar region of RNA2. This region is important as it has previously been reported to be deleted in FHV DI-RNAs. However, in the inoculum we do not observe deletions upstream in the RNA2 gene (for example 250^513), also reported to be deleted in previously characterized FHV DI-RNAs. Therefore, this dataset suggests that intermediate DI-RNAs with only a single region deleted between ~740–1220 are formed very early during virus passaging (within 3 days).

In passages P1-P2, less than 1.3% of the all the reads mapped to recombination events (**Table 1, Fig 2A**). Again, these occur throughout each of the genomic RNAs. However, we do begin to see events that have previously been characterized as forming DI-RNAs, such as 311^1104 and 301^1100, although these events are present at low levels (70 and 21 reads in Replicate 1 from a total of 8.2M reads mapped to the FHV genome) (**S1 Datafile**). In subsequent passages there was a rapid increase in the total proportion of mapped recombination events (peaking at 11.9% in P8R1) (**Table 1, Fig 2A**). In these later passages for each replicate, it can be seen that the most common recombination events are deletions that span two regions in each genomic RNA including: nts 300–940 and nts 1240–2300 of RNA1; and nts 250–510 and nts 740–1220 of RNA2, consistent with previous observations of FHV DI-RNAs[31] (**S1 Datafile**). However, the exact sites of the recombination events, while repeatedly observed over time in each replicate, varied between replicates and each had distinct ‘most popular’ species in the final passages **(Table 2**). In some instances we are able to find that a specific event is predominant in one replicate while at low levels in another. Overall, these trends in the frequency of recombination events throughout passaging reveal that once defective RNA species emerge during viral passaging they rapidly accumulate.

**Table 2:** Five most common events in each genomic RNA in the final passage of each replicate.

**Sample**	RNA1 Events	Count	RNA2 Events	Count
**Replicate 1**				
	301^1100	181,226	249^517	4620
	2643^2700	150,961	1086^1175	4228
	1350^2191	132,053	247^257	2239
	1243^2309	26,928	734^1233	1152
	2545^2685	26,574	727^1229	1097
**Replicate 2**				
	313^941	36,749	250^513	22,164
	2629^2644	36,332	736^1219	21,178
	2545^2685	24,858	249^517	6342
	342^1083	24,754	223^521	1167
	1245^2514	18,430	734^1233	1012
**Replicate 3**				
	1241^2298	236,140	249^517	3236
	317^945	67,911	242^525	1865
	1241^2305	49,348	778^1219	1722
	2545^2685	28,833	738^1219	1716
	344^915	16,480	1086^1175	352

The most common recombination events detected for each genomic RNA in passage 9 are indicated next to the number of reads that map over them. While similar regions are deleted, the exact recombination site varies slightly between each replicate.

Despite the predominance of certain recombination events in the later passages, there still remained a large number of infrequent events scattered throughout the viral genome. Many of these are observed only in one passage and not in subsequent passages. Again, these events likely correspond to stochastically generated RNA recombination events that form non-viable defective RNAs. We reasoned that analyzing these events would more accurately reveal the nucleotide preference of the FHV RdRp for RNA recombination as they were not subject to replicative selection (although they must be packaged by FHV particles), unlike the DI-RNAs. Therefore we extracted all recombination events occurring with fewer than 10 mapped reads throughout all FHV passages and replicates (20’723 unique events from a total of 11.6M possible permutations [8]) and counted the frequency of nucleotides found both up and down-stream of 5’ and 3’ recombination sites in the reference genome. As shown in **Fig 2C**, this revealed a preference for A’s 1-3nts downstream of 5’ sites, a preference for U’s 1-3nts upstream of 5’ sites, a weaker preference for a C 1nt up stream of 5’ sites, and an aversion to G’s 2nts both upstream and downstream of 5’ sites. Interestingly, an almost identical trend was observed for the 3’ sites. This trend was maintained also when analyzing only recombination events without ambiguity in the site of recombination (i.e. sites that lacked ‘fuzziness’ as reported by the *ViReMa* pipeline[32]). This result is similar to what we have previously reported[8]. However, here we provide a much larger dataset and analyze events that we can determine are not amplified in subsequent passages, providing greater confidence that these sites reflect the preference for RNA recombination at these sites rather than the selection of replicatively viable defective RNA species.

### Open reading frames are maintained in most DI-RNAs

Since many recombination events resulted in deletions of the viral genome, we were curious to see if the open reading frame (ORF) was conserved, as conservation of an ORF has frequently been observed to be a property of defective and defective-interfering RNAs[58]. Moreover, it has previously been shown that cloned DI-RNAs vectors containing eGPF in their putative ORFs do indeed express fluorescent protein[59] although it is not clear whether a functional ORF is essential for DI-RNA formation or propagation. In the earliest passages, only ~33% of deletions removed a multiple of 3 nucleotides (i.e. they thus conserved the ORF), as would be expected if deletion events occurred randomly throughout the genome. However, with continued passaging, there was a general trend toward conservation of the ORF for both RNA1 and RNA2 (**Fig 2D**), although this was not the case for all replicates. Specifically, while initially showing an increase in ORF conservation, replicate 2 of RNA2 showed a decrease in the conservation of the ORF after passage 4, in contrast to the other two replicates, and in fact dips below 33%. Closer inspection of individual recombination events shows that this trend is driven by three of the four most common recombination events in replicate 2 passages 4 to 9: 249^517, **736^1219, 250^513** and **249^519** (the latter three events in bold do not maintain the ORF). These events are observed in the other replicates, but at a much reduced frequency (no more than 1% of the total RNA2 recombination events for reps 1 and 3) (**S1 Datafile**). This indicates that DI-RNAs can indeed accumulate without a strict requirement for a functional ORF. However, this analysis only takes a single recombination event into consideration and the Illumina reads are not long enough (150bp) to resolve multiple deletions at the same time. Nevertheless, neither compensatory recombination events including small InDels that might restore the ORF, nor single nucleotide variants at putative stop-codons, were found.

### Conservation mapping illustrates emergence and accumulation of DI-RNAs

**Fig 3:**
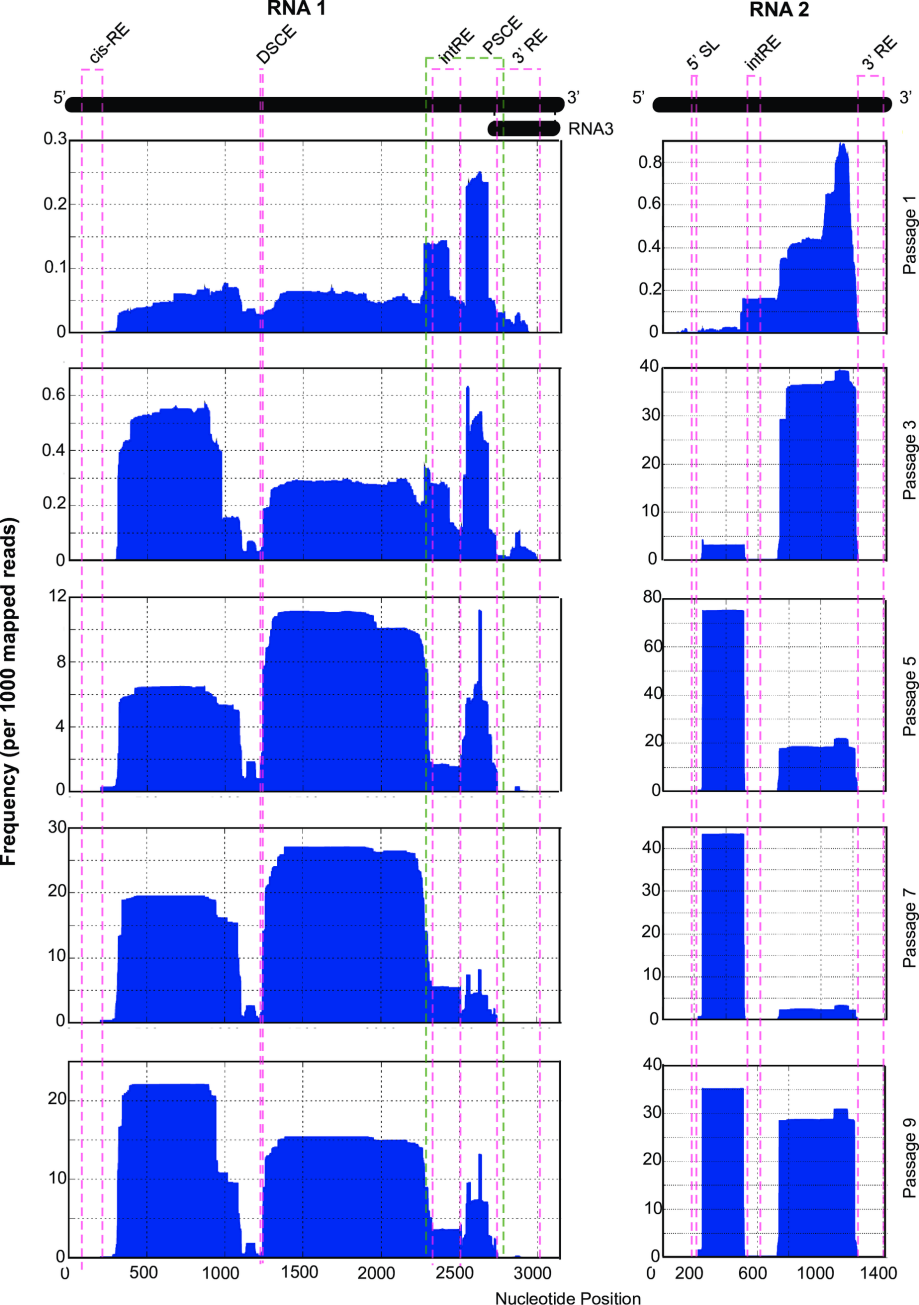
Recombination profiling of conserved regions in the DI-RNAs. Conservation map represents the frequency with which specific nucleotides in the FHV genome are deleted after recombination. Dashed lines indicate boundaries of functional RNA motifs. Odd numbered passages from replicate 2 are represented here. See **S3 Fig** for all replicates and all passages. *Cis-RE*: cis-Regulatory Element; *DSCE/PSCE*: Distal/Proximal Subgenomic Control Elements; *intRE*: internal Response Element; *3’ RE*: 3’ Response Element; *5’ SL*: 5’ Stem Loop

Using *ViReMa*, we were able to calculate the frequency with which each nucleotide was deleted, revealing areas of the viral genome that are conserved during serial passaging and required for DI-RNA replication. We plotted these data to generate recombination profiling maps for each RNA of FHV throughout passaging (**Fig 3**and **S2 Fig**). In the first passage, there is a relatively even distribution of nucleotide deletions along the whole length of the genome with the exception of two frequently excised regions in the 3’ end in RNA1 due to two common recombination events in each of the replicates: 2545^2685 and 2277^2435. By passage 3 the deletions along the genomic landscape begin to be ‘*sculpted’* whereby certain regions are deleted with a greater frequency than others. Passages 5, 7, and 9 were sculpted further revealing three major regions that were deleted in RNA1 and two in RNA2. Interestingly, for both RNA segments, while a range of deletions and rearrangements are generated during early passages, only the deletions that maintain regulatory and control elements are amplified during continued passaging, as previously observed in FHV [32]. These include the 5’/3’ UTRs and internal response elements (intRE) of both genomes, as well as the Proximal- and Distal-Subgenomic Control Elements (PSCE and DSCE) in RNA1 (**Fig 3**), which correlates to the findings that these regions are important and required for RNA replication and encapsidation [29, 30, 32, 60–64].

### MinION nanopore long-read sequencing of Flock House virus

**Fig 4:**
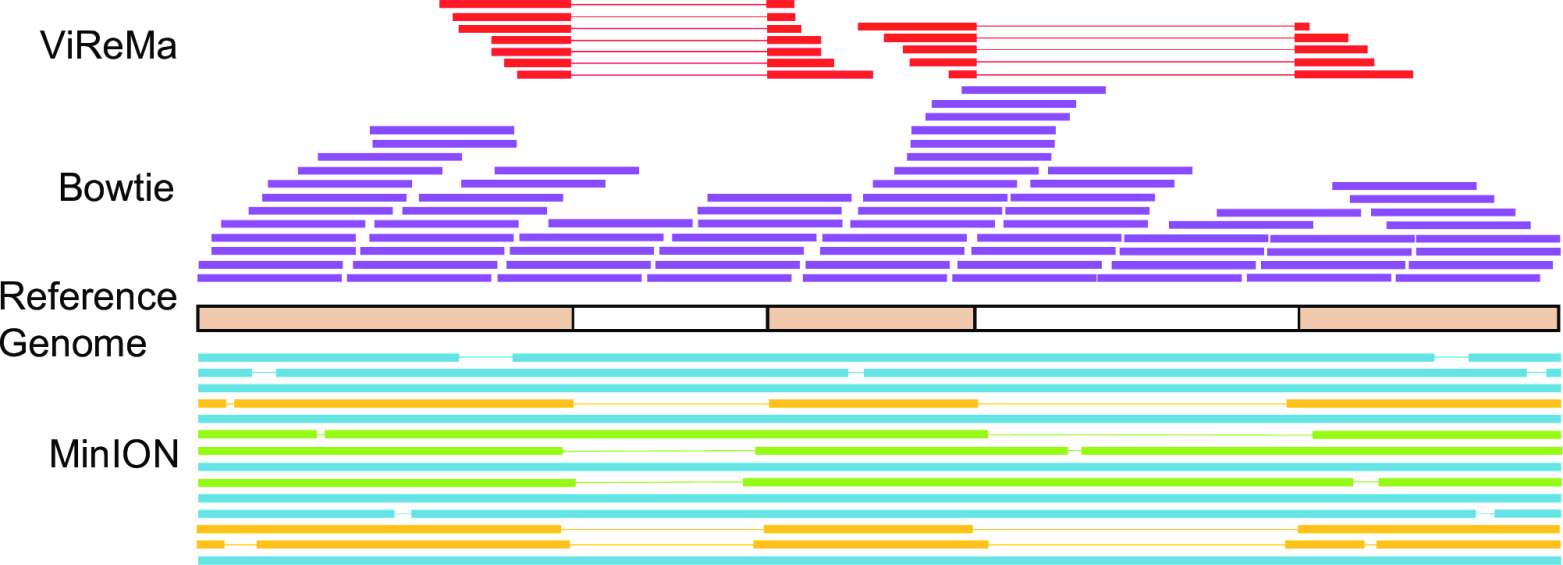
Comparison of Illumina HiSeq to Oxford Nanopore MinION read data. Example of how reads generated from the Illumina HiSeq would map to a reference genome. The *Bowtie* alignment is able to map 150bp reads along the reference with a relatively even coverage distribution. Unmapped reads are then aligned with *ViReMa* to accurately identify recombination events. The low error rate of high-throughput sequencing allows us to precisely define the boundaries of the junctions. Below the reference genome is an example of how reads generated from Oxford Nanopore Technologies’s MinION sequencing would map to a reference genome. The MinION is able to generate full length reads at the expense of a high error rate, which is ~7%. Full length analysis allows us to determine what recombination events a genome contains. Due to the error rate the exact boundaries of the recombination event are imprecise.

**Fig 5:**
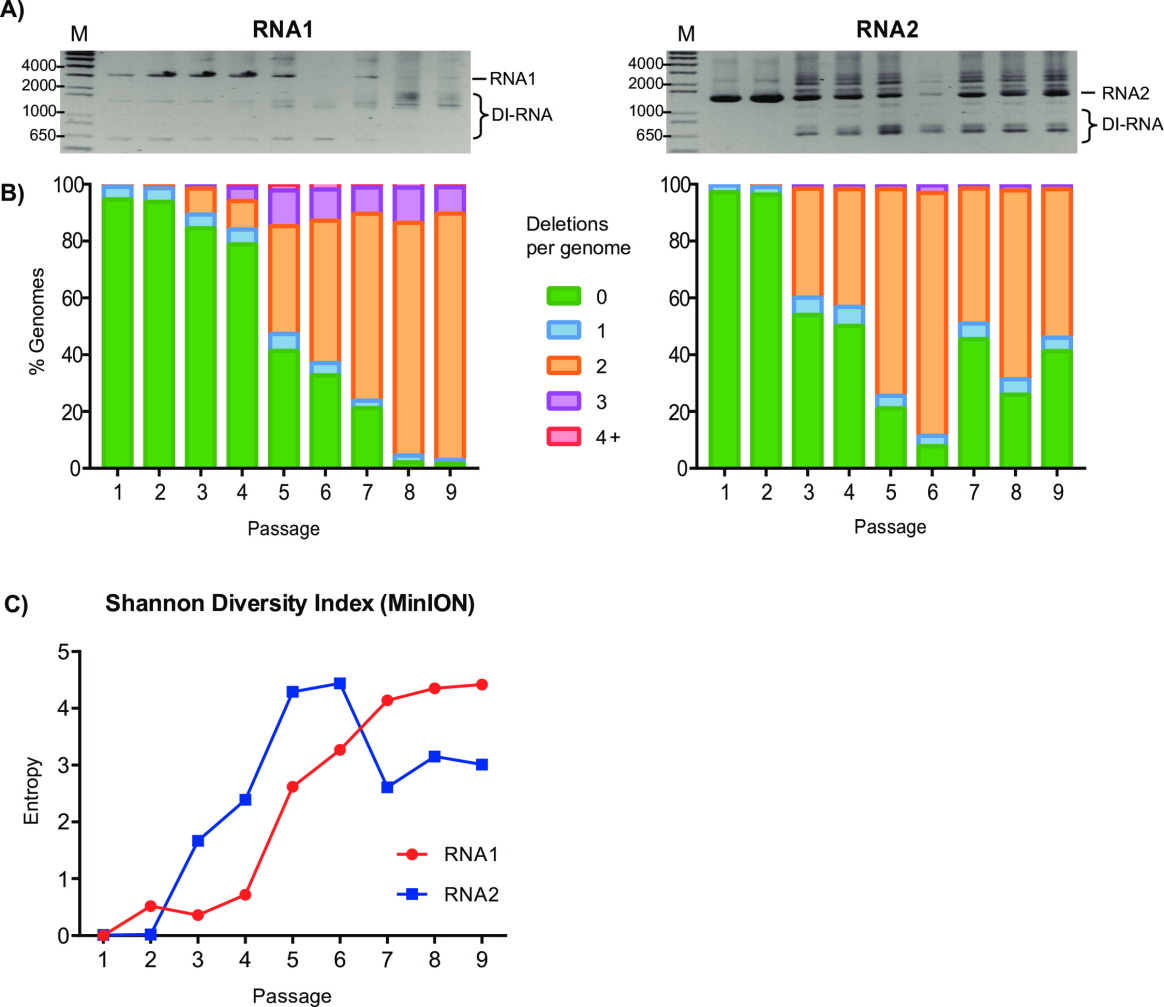
The frequency of deletions in the FHV genomic RNAs found by MinION nanopore sequencing. **(A)** Gel electrophoresis analysis of cDNA copies for RNA1 and RNA2 for each passage in replicate 2 shows full-length viral genomic RNAs in early passages with increasing quantities of smaller defective RNAs bands in later passages. **(B)** Full length genomes were sequenced with the MinION and the number of deletions per genome were counted for each passage. Due to the higher error rate of the nanopore data only deletions ≥25nts were counted. **(C)** The Shannon Diversity Index of all RNA1 or RNA2 genes characterized with MinION nanopore sequencing is shown for each passage.

The short-read ClickSeq data provide in-depth and high resolution details of individual recombination events. However, in order to determine the correlation of these events over time, we used long-read ONT nanopore sequencing, which can characterize full-length wild-type and defective genomes (**Fig 4**and **S4 Fig**). We reverse transcribed and amplified both RNA genes using primers specific to the 5’ and 3’ UTRs of RNA1 and RNA2 from all passages of replicate 2 to obtain cDNA that could be barcoded and analyzed using protocols for 2D sequencing on the ONT MinION. The ClickSeq data shows that these regions were highly conserved during passaging (**Fig 3**), therefore we were confident using template-specific primers to these regions would capture both the full-length wild-type virus genomes as well as any defective RNAs. After PCR amplification, we analyzed the MinION cDNA libraries using agarose gel electrophoresis to observe the distribution of cDNA fragments and ratios of full-length to defective RNA genomes (**Fig 5A**). While the cDNAs from the early passages are predominantly of the expected size for full-length RNA genomes, later passages contain an array of band sizes. This shows that in the early passages the full-length genome is the predominant species while in later passages the truncated version becomes predominant. We also observe species appearing to be larger than RNA2. It is possible that some of these species correspond to RNA2 homodimers or other complex rearrangements that have previously been observed[65] and would result in an increased molecular weight. Evidence of RNA1 homodimers (3107^1), RNA2 homodimers (1400^1), and RNA2 to RNA3 heterodimers (1400^2720) can also be found in the ClickSeq data (**S1 Datafile**).

We pooled the amplified cDNAs from each sample at equimolar ratios and loaded the pooled, barcoded library onto a MinION MkIB device using an R9 flowcell as per the manufacture’s protocol. We ran the standard protocol for obtaining 2-dimensional reads using the MinKNOW control software and collected nanopore reads for approximately 36 hours, upon which the quality and yield of reads dropped substantially. We obtained a total of 169’814 reads, of which 46’183 passed the default ONT filter and were successfully demultiplexed. This yielded between 2688 and 8815 full-length reads per passage and corresponds to approximately 0.1 Gigabases of sequence information. This would correspond to ~80’000 1x150bp Illumina reads per sample, assuming even coverage. With this depth, we can build up a comprehensive picture of the full-length genomic landscape of the viral samples, allowing us to resolve DI-RNA species even if they were present at less than 1% of the total viral genomic population.

The long-read nanopore sequencing data were aligned to the full-length FHV genome using the BBMAP suite (https://sourceforge.net/projects/bbmap/). This pipeline tolerates large insertions and deletions in the long-reads, thus allowing us to characterize the overall topology of the defective RNAs. We mapped between 94 and 97.5% of the MinION reads from each passage to the FHV reference genome (**Table 3**). An example of reads aligned to FHV RNA2 is shown in **S4 Fig**. The error rate of aligned reads, including single nucleotide mismatches and small InDels, was determined from alignment pileup files. Consistent with recent reports [66], we found the overall modal and mean error rates for all mapped position was 5.0% and 6.3% respectively, with 95% of the sites having an error rate better than 13.6% (N95 value = 0.864). A histogram of error rates for all mapped positions across all 9 datasets is shown in **S5A Fig**. Due to the large number of small InDels generated during nanopore sequencing [43], we also determined the frequency of deletions and insertions of different lengths (**S5B and S5C Fig**). This shows that small InDels are frequent, but fall quickly in abundance with increasing length. 99.8% and 99.9% of all MinION deletions and insertions respectively were shorter than 25nts. Therefore, we considered only deletions and insertions of at least 25nts to be likely to be *bona fide* InDels present in the original viral RNA and corresponding to recombination events comprising defective RNA species rather than a sequencing error.

**Table 3:** Nanopore sequencing quantifies the number of deletions found per full-length cDNA.

	Passage 1	Passage 2	Passage 3	Passage 4	Passage 5	Passage 6	Passage 7	Passage 8	Passage 9
**Total Reads**	**3842**	**5398**	**3055**	**5013**	**7675**	**3710**	**3413**	**2688**	**8815**
**RNA1**	**2118**	**2686**	**1426**	**2392**	**3703**	**2038**	**1991**	**1562**	**5387**
**# of deletions: 0**	*2006*	*2519*	*1206*	*1886*	*1532*	*667*	*422*	*33*	*85*
**1**	*94*	*131*	*68*	*127*	*223*	*89*	*53*	*38*	*74*
**2**	*13*	*29*	*131*	*239*	*1405*	*1021*	*1310*	*1280*	*4674*
**3**	*5*	*6*	*19*	*111*	*464*	*225*	*184*	*193*	*499*
**4**	*0*	*1*	*1*	*26*	*66*	*30*	*19*	*18*	*48*
**5**	*0*	*0*	*1*	*3*	*10*	*6*	*3*	*0*	*7*
+	*0*	*0*	*0*	*0*	*3*	*0*	*0*	*0*	*0*
***R*, MinION vs ClickSeq**	0.08	0.02	0.38	0.30	0.63	0.68	0.64	0.27	0.52
**RNA2**	**1515**	**2388**	**1455**	**2336**	**3743**	**1595**	**1299**	**989**	**3207**
**# of deletions: 0**	*1473*	*2301*	*786*	*1170*	*791*	*124*	*592*	*257*	*1325*
**1**	*36*	*64*	*89*	*158*	*164*	*59*	*71*	*53*	*150*
**2**	*6*	*22*	*558*	*968*	*2723*	*1364*	*617*	*658*	*1678*
**3**	*0*	*1*	*21*	*38*	*62*	*42*	*19*	*17*	*51*
**4**	*0*	*0*	*1*	*2*	*3*	*6*	*0*	*4*	*3*
+	*0*	*0*	*0*	*0*	*0*	*0*	*0*	*0*	*0*
**R, MinION vs ClickSeq**	0.09	0.01	0.55	0.85	0.73	0.59	0.63	0.38	0.56
**Unmapped Reads**	**209**	**324**	**174**	**285**	**229**	**77**	**123**	**137**	**221**

The number of demultiplexed nanopore reads passing quality filters are shown for each sample. These were mapped end-to-end to the FHV genome using the BBMap suite, allowing for large deletions and insertions, which were counted using the CIGAR string from the output alignment SAM file. The Pearson correlation coefficients of these events to those found using ClickSeq were also calculated. The number of reads mapping to FHV RNA1 and FHV RNA2 are indicated along with the number that contain 0–5 or more deletions of at least 25 nts. Only a small number (<5%) remained unmapped.

### Long-read nanopore data characterize defective RNAs and the correlation of deletions

The nanopore data reveals the presence and frequency of large deletions and insertions within defective RNA genomes. From these, we can reconstruct the population of either full-length or defective RNA genomes present in each of the viral passages (annotated as described in the *Methods* section). The full table of characterized defective RNAs and their frequencies in each passage is detailed in **S2 Datafile**. In total, we found 6030 and 3639 unique defective RNAs of RNA1 and RNA2 respectively throughout all passages.

The frequency of individual recombination events found in both the ClickSeq data and the MinION data were compared and correlation coefficients calculated (**Table 3**). The correlation in the earliest passages was poor, due to the low abundance of events in both datasets. However, later passages correlate well with Pearson coefficients reaching 0.85. This is important as it demonstrates that the frequency of recombination events was not biased during cDNA amplification of the full-length or defective viral genomes. Similarly, the Shannon Entropy indices increase during passaging (**Fig 5C**), consistent with those from the ClickSeq data.

The number of deletions in each passage in RNA1 and RNA2 are given in **Table 3**and illustrated in **Fig 5B**. The earliest passage contains very few deletions. In passage 1, 95.8% of the reads map to the full-length genome in its entirety. With subsequent passages, the number of reads containing deletions increases, reaching a plateau at passage 8 with 76.0% of the reads containing two deletions and 8.2% containing three deletions. DI-RNAs (e.g. 1_317^1091_1242^2301_3107) are easily identifiable as early as passage 2 (**S2 Datafile**) and match well with the expected identities based on our ClickSeq results and previous studies[8, 31]. By the final passages these species predominate, leaving only a small percentage of full-length wild-type viral RNAs.

While we can readily identify mature DI-RNAs containing two or more deletions, few single-reads contain just one deletion (<6%) in all of the passages. Moreover, individual species are rarely observed again in subsequent passages (**S6 Fig** and **S2 Datafile**). Importantly, most of these single events do not delete the expected regions common to FHV DI-RNAs. Therefore, they may either correspond to sequencing artifacts, or transient defective RNA species generated due to stochastic RNA recombination, similar to the low-frequency events observed in the ClickSeq datasets. In later passages (beginning at passage 3), we do begin to see the presumptive intermediates (e.g. 1_317^1091_3107 or 1_1242^2301_3107) of mature DI-RNAs (e.g. 1_317^1091_1242^2301_3107). However, this is after the mature DI-RNAs were first observed, and after the point at which DI-RNAs have begun to accumulate. Indeed, in passages 2 and 3 respectively, mature DI-RNAs make up 1% and 30% of the viral population while the singly-deleted intermediates make up 0% (unobserved) and 3%. Together with the observation of rare DI-RNAs in the inoculum with the ClickSeq recombination analysis, these data indicate that single-deletion species do occur early during passaging, but remain poorly abundant and do not accumulate. In contrast, mature DI-RNAs are observed to rapidly accumulate between passages, indicating that they possess a replicative advantage above both wild-type viral genomes and intermediate defective RNA species.

### Complex rearrangements are observed by MinION and confirmed by ClickSeq

In addition to deletions, a small number of defective RNAs first appearing at passage 5 contained insertions. Interestingly, the majority of these comprised short insertions of ~200 nucleotides that were found in first 300nts of the MinION reads and were mapped between nts 19 and 20 of RNA1. In each case, these inserts corresponded to nts 2300–2513 of RNA1. This region corresponds to an internal response element (intRE) of the Proximal Sub-genomic RNA Control Element (PSCE) previously identified as being essential for FHV RNA replication and conserved in DI-RNAs species [60]. The most common deletion in the DI-RNAs in this region of RNA1 for the final passages are from 1242 to 2301, which retains the intRE. However, there are also a large number of deletions ranging from 1245 to 2514, which would delete the essential intRE. Closer inspection of the MinION data reveals that the majority of these reads (>90%) that contain the 200nt intRE insertion concomitantly contained deletions from 1245–2514, indicating that these two events are correlated.

The ClickSeq data also shows a frequent recombination event, 2513^21, which appears first in passage 4 and is among the 10 most common events in the final 5 passages (**S1 Datafile**). This matches precisely the 3’ junction site of the insertion event detected in the MinION data. However, the event 20^2300 corresponding to the 5’ junction site was not detected in our initial *ViReMa* analysis of the ClickSeq data as this would have required a search seed length of less than 20 nts. Repeating the *ViReMa* analysis using a seed length of 17 does indeed reveal the presence of the 20^2300 recombination event. This event is rarely observed in either of the other two replicate ClickSeq data (7 and 31 total reads across all passages of replicates 1 and 3 respectively). These data indicate that in a number of defective RNA genomes, the intRE element has been deleted and subsequently re-inserted at the 5’ end of the defective RNA genome. As the intRE element is required for regulation of RNA replication, presumably this maintains the ability for this highly-rearranged defective RNA to replicate.

### MinION nanopore sequencing reveals the emergence, diversity and evolution of DI-RNAs

**Fig 6:**
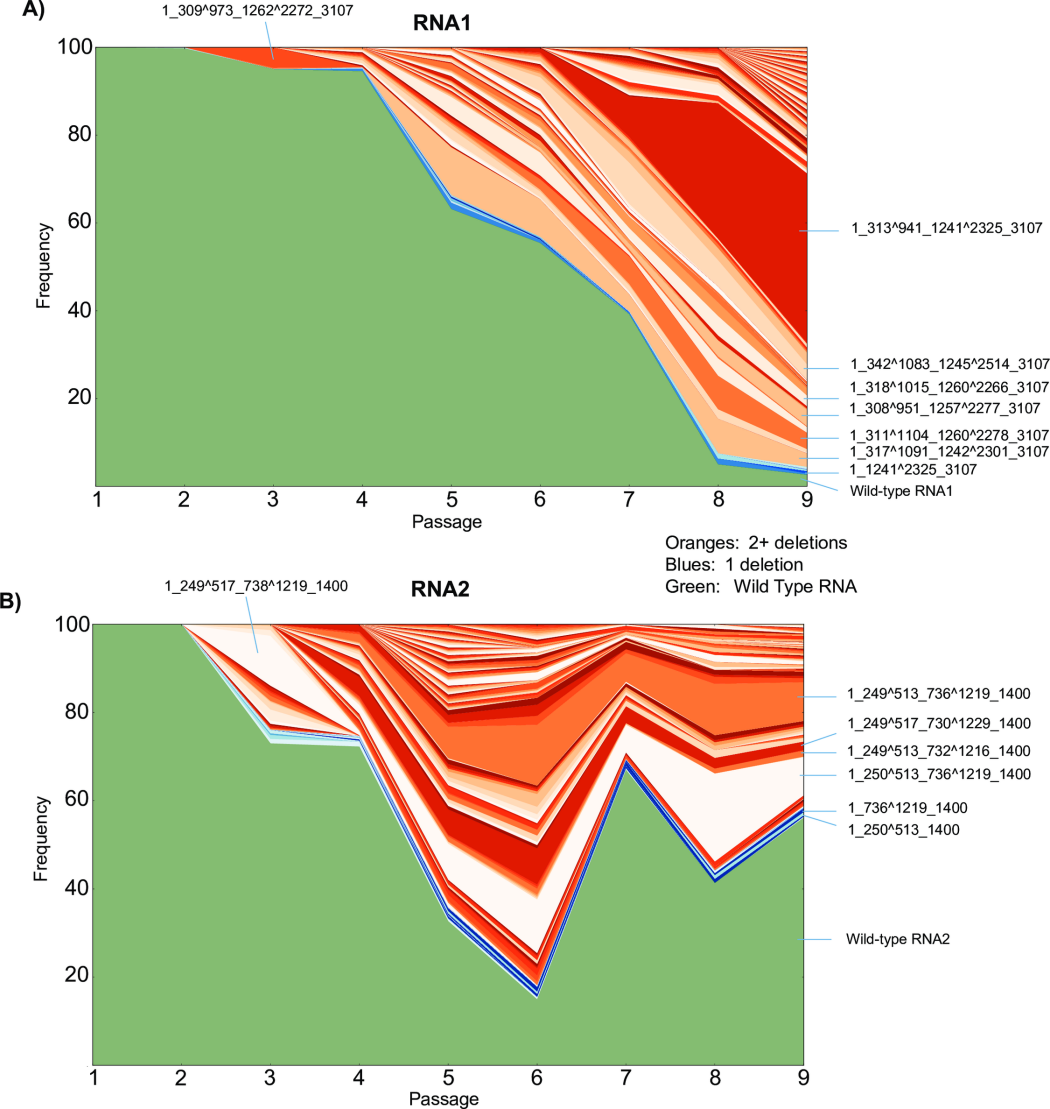
**Evolutionary pathways of full-length RNA genomes are illustrated using stacked-area plots** for **(A)** RNA1 and **(B)** RNA2. The passage number is indicated on the x-axis and the stacked frequencies of each detected defective RNA is shown in the y-axis. Each non-contiguous color represents a specific genome characterized by MinION nanopore sequencing. Wild-type genomes are colored green, genomes with one deletion are colored in shades of blue, and genomes with two or more deletions are colored in shades of orange (using the same color scheme as in **Fig 5B**). Raw data and annotations are in **S2 Datafile**.

These data provide a comprehensive overview of the different species of defective RNAs that are present during viral passaging. Illustrating such a complex set of data is a challenge as each sample contains a large number of genome arrangements (6030 and 3639 for RNA1 and RNA2 respectively) and frequencies of these species vary substantially over time. We found that illustrating these data as a stacked area plot gave the most informative summary of the changes of the many different type of DI-RNA species over time. Due to the moderate error-rate of the nanopore read data, the exact identification of a recombination event and thus annotation of that genome may be incorrect. This would result in an over-estimation in the potential number of unique structural variants. Therefore we filtered datasets by requiring genomes to be represented by three or more reads. While removing a lot of noise, this has the drawback that we might be losing rare defective RNAs. Stacked area plots for genomes represented by three or more reads are shown in **Fig 6**. The stacked area plots for the unfiltered datasets are shown in **S6 Fig**. This representation reveals key components of the evolution of the DI-RNA species.

The stacked area plot for RNA1 (**Fig 6A**) shows that the composition of DI-RNAs in the viral population changes over time and new species appear at each passage. For example, the most abundant defective RNA1 species in passage 5 is ‘*1_317^1091_1242^2301_3107*’ but reduces in relative frequency in later passages. The most abundant species in the final passage 9 is ‘*1_313^941_1241^2325_3107’*, which appears at low levels as early as passage 2, but does not begin to accumulate until passage 6 (**S2 Datafile**). Why this DI-RNA only begins to accumulate at later passages despite being present in the early passages is not clear. The ‘complex DI-RNA’ that deletes the PSCE in RNA1 referred to in the previous section (*‘1_342^1083_1245^2514_3107’*) is also observed (annotated in **Fig 6A**) first appearing at passage 5.

As can be seen in the stacked area plot for RNA2 (**Fig 6B**), the general composition of DI-RNA species is established at passages 4–5. Subsequently, the relative frequencies of the DI-RNA fluctuate but the overall diversity changes little with few new species appearing after passage 4. This is also observed when calculating the Shannon Diversity index (**Fig 5B**) whereby entropy reaches a maximum at passage 5 and decreases thereafter. Interestingly, this range of fluctuations resemble the sinusoidal patterns of DI-RNA abundance that have been observed in other studies of RNA viruses where the ratio of the frequency of DI-RNAs to wild-type genome has been measured through longitudinal studies[67].

### Specific infectivity correlates with abundance of defective RNAs

**Fig 7:**
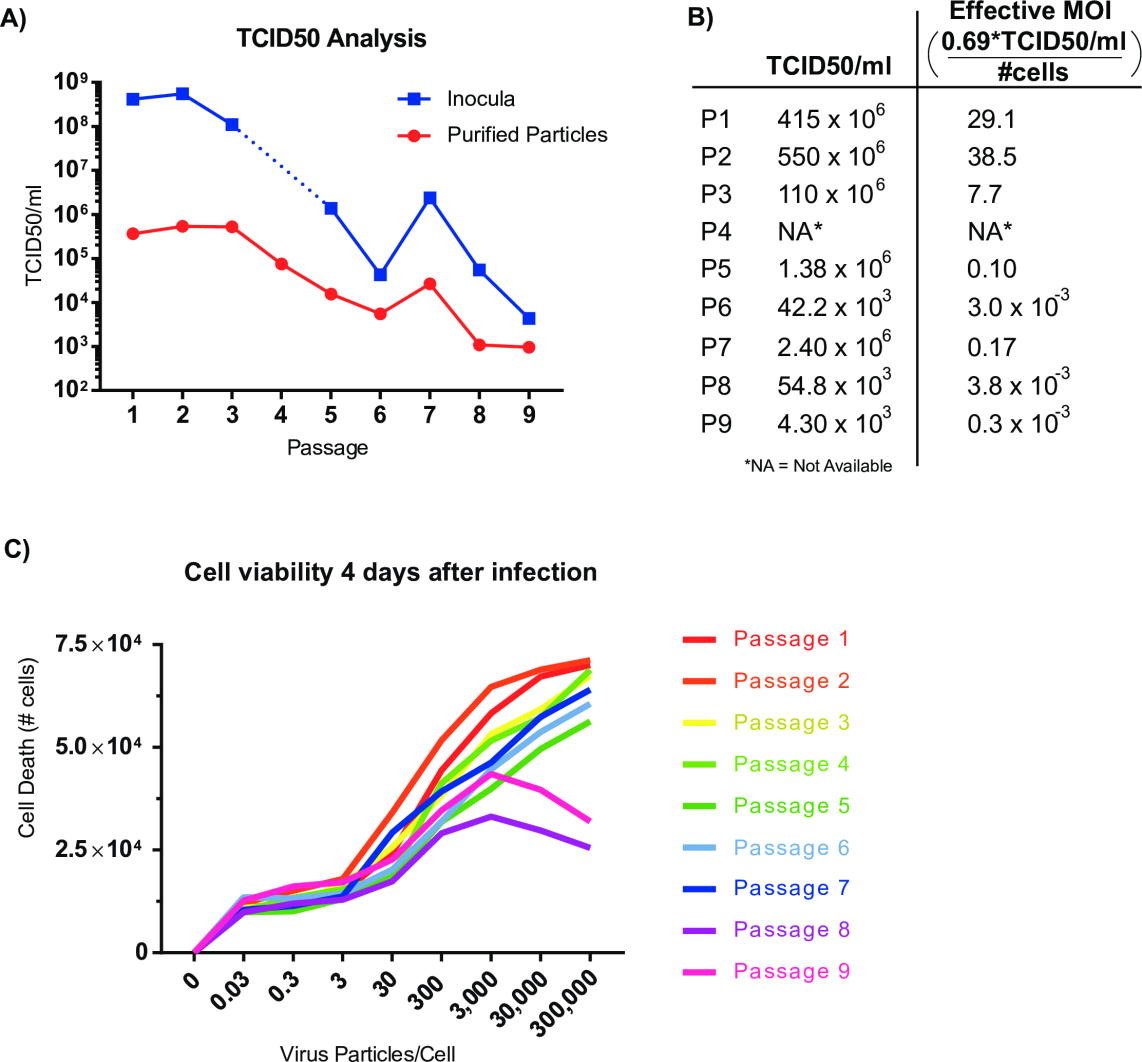
TCID50, specific infectivity and cytopathic effect (CPE) of each passage for replicate 2. **(A)** The 50% tissue culture infectious dose (TCID50) of each passage of replicate 2 is shown (inocula used in serial passaging in blue squares). Additionally, virus was purified and quantified by OD_260nm_ before infection allowing normalization particles per cell across all replicates for further TCID50 analysis (in red circles). **(B)** TCID50/ml values were used to calculate effective MOI values during serial passaging. *Passage 4 sample was not available. **(C)** 10^5^ cells were infected with quantified and serially diluted virus. The number of virus particles per cell plated is indicated on the x-axis. After 4 days of infection, the remaining number of cells was analyzed by flow-cytometry to count remaining viable cells. Cell death is indicated by the difference in count compared to non-infected wells (0 particles/cell). Each line indicates a different passage from replicate 2 as indicated in the color key.

The reduction in full-length infectious viral genomes and the accumulation of defective RNAs during passaging is likely to correspond to a decrease in the specific infectivity of the virus samples. To determine the effect of defective RNAs characterized by combined ClickSeq and nanopore sequencing of replicate 2 upon specific infectivity, we performed 50% tissue culture infectious dose (TCID50) assays for each passage 1–9 for both the original inoculum used to infect each sample and for the particles purified from each passage [51]. The TCID50 assay is used to determine the dose required to give a 50% chance that cells in culture will be successfully infected as determined by CPE and is typically used to determine viral titer and the effective MOI of the inocula transferred from passage to passage. The results from the TCID50 assay for each passage are shown in **Fig 7A and 7B**. We found that the TCID50 value (and thus PFUs(Plaque Forming Units)/ml) drops considerably during passaging by over four orders of magnitude. The corresponding effective MOI (PFUs per cell) also drops from 38.5 to 0.0003 during passaging (**Fig 7B**).

To determine whether the drop in effective MOI was driven by reduced total particle yield or from reduced specific infectivity (i.e. virus particles per PFU), we also performed TCID50 analysis of our purified and quantified virus stocks (described in *methods*). This allowed us to determine and normalize the number of virus particles delivered per cell between each passage. As the particle-per-PFU ratio has previously been estimated at 300–400 particles-per-PFU [26, 52] we setup our assay beginning with 300’000 particles per cell in 96 well format and performed 8 10-fold serial dilutions. In this assay, we found that the number of viral particles required to induce CPE decreased by over 400-fold during passaging (**Fig 7A**) with a trend very similar to that for the unpurified inocula. Together, these data indicate virus specific infectivity drops with a corresponding increase in the defective RNA population. There was an exception at passage 7 where TCID50 actually increased ~5 fold from the previous passage. This could be correlated to our observation of a decrease in the amount of defective RNA2 species in the MinION analysis (**Figs 5B** and **6B**).

We further characterized each well of the TCID50 assay of our purified particles using flow-cytometry to give a quantitative assessment of cell survival and death in response to virus dose. We calculated the number of live cells that remained after infection at each dilution and for each passage (**Fig 7C and S7 Fig**). We observed a reduced overall CPE in later passages at the highest virus dose as well as an increase in the number of viral particles require to induce the same amount of CPE (**Fig 7C)**. Together these trends reflect a reduced specific infectivity during viral passaging, in agreement with our TCID50 assays. Interestingly however, for the highest particle concentrations in passages 8 and 9, we saw less cell death at the highest doses (300,000 and 30,000 particles per cell) than for cells infected with the same inoculum (and therefore same ratio of full-length to defective RNAs) but at a lower dose (3,000–30 particles per cell). This observation indicates the protection of cells from infection and/or CPE when supplied with a large dose of viral particles that contain a large proportion of DI-RNAs.

## Discussion

In this manuscript, we sought to provide a thorough and comprehensive analysis of the frequency and identity of recombination events present during the serial passaging of Flock House virus in cell culture in order to elucidate the pathways and mechanism of DI-RNA emergence and evolution. We began with a homogenous inoculum derived from plasmid cDNAs of each of the FHV genomes. In the inoculum and in the early passages, we find a wide range of low-frequency recombination events corresponding to deletions and duplications that are dispersed through-out the viral genomic RNAs. We can be confident that these species do not constitute sequencing artifacts as we made our RNAseq libraries using ‘ClickSeq’[35] that has previously been demonstrated to reduce artifactual recombination in RNAseq data to fewer than 3 events per million reads. Further confidence in the low rates of artifactual recombination in our study is provided internally by inspecting the numbers of inter-RNA recombination events (RNA1 to RNA2 and *vice versa*), which are always low. Furthermore, the majority of the detected inter-RNA recombination events correspond to genomic RNA hetero- and homo-dimers, which have previously been characterized as replication intermediates [65].

Within only 2–3 passages, however, deletion events similar to those previously observed in DI-RNAs appear in all three passaging replicates. In subsequent passages, these recombination events begin to accumulate rapidly so as to predominate over full-length viral RNAs. This observation of the emergence of DI-RNA species, followed by their rapid accumulation is consistent with existing theories on the evolution of DI-RNAs that postulate that a wide range of potential DI-RNA species are generated by non-programmed RNA recombination and that only a handful are successfully replicated and thus accumulate[9]. While the short-read data provide high-resolution characterization of individual recombination events, it is through the use of the Oxford Nanopore Technologies (ONT) MinION that we are able reconstitute the complex full-length genomic landscape of FHV during passaging and determine the relative abundances of the genomic RNAs in each passage. As a result, we were able to determine that by the final passage only ~2% of the mapped reads are full-length viral RNA1, which corresponded with a large reduction in specific infectivity. Additionally, the nanopore data revealed complex rearrangements of RNA genomes, including the excision of an entire functional RNA motif and its reinsertion in the 5’UTR of RNA1.

The variation in recombination boundaries of the DI-RNAs suggests that a range of deletions can be tolerated. However, it is important to note that while each replicate is its own distinct lineage, each replicate passaging experiment was derived from the same initial inoculum. We chose to design the experiment this way to determine if the same DI-RNAs would be generated independently or if completely different deletions would arise and be selected for even though the environmental conditions are practically the same. Here, we observe the latter (**Table 2**). Few of the RNA1 recombination events observed in the inoculum are observed again in subsequent passages. Additionally, even though we found the event ‘738^1219’ in RNA2 the inoculum, this was not the final predominant species in any replicate. Therefore, the evolution of DI-RNAs was not pre-determined by the presence of rare DI-RNA species in the common inoculum (a founder-effect), but rather by the selection of well-replicating DI-RNAs that arose later during serial passaging. Nonetheless, the final recombination events are highly similar between replicates and to previous reports from different laboratories. Therefore, this indicates that either the DI-RNAs have emerged due to a common mechanism of formation, the presence of a common selectivity filter, or both.

In addition to providing a thorough analysis of the pathways of defective RNA formation and evolution, there are two unexpected and critical observations made through this study. First: while we observe a wide range of recombination events early on during passaging, only a limited number of events are subsequently amplified and later *define* DI-RNAs. Moreover, these limited sets of events are similar between replicates, and to previous studies. This suggests that while a large pool of potential defective RNAs are generated, only a small number are capable of accumulating. Secondly: we do not observe the amplification of DI-RNAs with only one deletion. In contrast, ‘mature’ DI-RNAs accumulate rapidly. Nonetheless, we do find evidence for intermediate DI-RNAs as early as the inoculum sample. This indicates that intermediate defective-species are either non-competitive and do not accumulate or are not formed as a pre-cursor to mature DI-RNAs. These two observations provide important insights into the potential mechanisms of DI-RNA emergence and evolution.

While there is a strong selection pressure for DI-RNAs to retain essential functional genomic elements, it is also postulated that a shorter defective RNA would be replicated more quickly and thus more competitively [10]. In our analysis, while the ~250–550 deletion in RNA2 (for example) is very common we do not observe the accumulation of deletion events that are smaller than this (e.g. 300–450). This is despite the fact that we can detect and observe such species in low frequencies both in early and late passages, suggesting that they are indeed generated but are not selectively amplified. This may in part be due to selecting for DI-RNA genomes that are as small as possible, while retaining the minimal amount of genetic material to form functional genetic elements. However, it may also be the result of a negative selection pressure or restrictive barrier that is released only after excising specific portions of the viral genome. An example of this scenario has been demonstrated for tomato bushy stunt virus (TBSV) associated DI-RNAs whereby deletion of a translation enhancer functional element removes the competition between translation and replication, thus favoring replication of the smaller DI-RNA[68]. Therefore, the final structure of DI-RNAs may depend both on the retention of essential functional RNA elements, as well as the removal of restrictive barriers that attenuate RNA replication.

One model for the evolution of DI-RNAs is through the step-wise accumulation of deletion events through a series of individual recombination events[68]. The MinION data reveals that the defective RNAs that accumulate (rapidly over the course 2–3 passages) contain multiple deletion events. However, we do not see the rapid accumulation of the intermediate DI-RNAs, despite evidence for their presence early in viral passaging. This suggests that the mature DI-RNAs have a competitive advantage over their presumed intermediate precursors. If this is the case, the multiple deletions may function epistatically either through an undefined cooperative/additive mechanism or through the release of multiple restriction barriers, as proposed above. If multiple restriction barriers are required to be excised for the formation of DI-RNAs, small or multipartite RNA viruses, such as FHV or influenza[17], may therefore generate DI-RNAs more readily by requiring fewer intermediate steps than long, monopartite RNA viruses. Moreover, if intermediate defective RNAs fail to accumulate, this reduces the likelihood that mature DI-RNAs can subsequently be generated and may place substantial limitations on the ability of some viruses to generate DI-RNAs altogether.

An alternative reason for the rarity of precursor/intermediate defective RNAs is that the mature DI-RNAs are generated in one single event. We are yet to determine the molecular mechanism of recombination that leads to DI-RNA formation. Both template-switching, secondary-structure jumping, and non-replicative mechanisms have been proposed, and indeed these mechanisms need not be mutually exclusive. Our observation of nucleotide preferences at recombination junctions (**Fig 2C**) may arise through any of these potential mechanisms. Alternatively, it is possible that multiple reassembly/deletion events occur in a single step, in a manner reminiscent of chromosome shattering (chromothripsis)[69]; or ‘*virothripsis’*. Within the confined invaginations of the mitochondrial membranes that form the replication factories of RNA viruses such as FHV[70], the fragmentation the RNA virus genome followed by incorrect re-stitching of these genome pieces, either through forced-copy choice template switching or a non-replicative mechanism, could create the DI-RNAs observed here including the complex rearrangements observed for RNA1.

A defective-*interfering* RNA is a defective RNA that has the ability to compete with or otherwise attenuate the replication and proliferation of the wild-type helper virus. In our study we demonstrate that the viral swarm, even after only a few passages, is replete with many varieties of defective RNAs. With a single sequencing experiment, we would not be able to determine whether these defective RNAs are accumulating, diminishing or make-up a static component of the viral quasi-species. However, as we perform serial passaging with sequential sequencing experiments, we can determine which defective RNAs are accumulating (for example the ‘mature’ DI-RNAs) and which are not (e.g. the putative intermediate, or ‘immature’ defective RNAs). It would be impractical to validate each of the many hundreds of detected defective RNA species with molecular virological experiments to determine whether they truly can attenuate or interfere with wild-type virus replication and to therefore categorize that species as a defective-*interfering* RNA. Indeed, we cannot exclude the possibility that multiple DI-RNAs act co-operatively within the viral quasi-species and are mutually dependent upon one another. However, the demonstration here of an accumulation during serial passaging is strong evidence that these species are *interfering*, as their accumulation essentially dilutes the pool of wild-type functional virus. With this in mind, we believe it would be suitable to describe the mature defective RNAs as defective-*interfering* RNAs (DI-RNAs), and the ‘immature’ only as defective RNAs.

It is remarkable that FHV is able to maintain a viable infection despite being burdened with such a gross excess of DI-RNAs in the final passages presented here. By performing TCID50 assays of the original inocula used between each of the passages and of the particles purified from each passage, we show that there is a dramatic reduction in specific infectivity during passaging corresponding with the increase in the DI-RNA content. DI-RNAs have generally been demonstrated to arise at high MOIs, as was the scenario with our first passages. However, our calculated MOI drops rapidly after DI-RNAs have formed to levels that might be expected during typical *in vivo* viral passaging scenarios. However, in this experiment we were actually passaging a large number of virus particles between cells, but with a low specific infectivity. It is also interesting to observe that for the final passages there appears to be a protective property of the DI-RNAs as determined by flow-cytometry, but only when administered at the highest doses corresponding to over ~30,000 particles per cell. However, the role that DI-RNAs might play *in vivo* is not clear, as these very high doses may not be physiologically relevant. DI-RNAs for FHV similar to the ones described here have been observed to arise in experimental fruit fly infections [33] and so the mechanism of formation and/or selection is likely to be similar in cell culture and *in vivo*. However, whether RNA viruses such as FHV have evolved to favor the spontaneous formation of DI-RNAs and if so whether these DI-RNAs play an important role in modifying the life-cycle of the virus, is yet to be determined.

## Supporting information

S1 Fig Percentage of ClickSeq reads mapping to host RNA through each passage and replicate. Percent mapping is calculated by the frequency of reads that mapped to the host genome compared to all processed reads.
(PDF)

S2 Fig Scatter plots comparing frequency of unique recombination events found in replicate ClickSeq libraries of P7R2. Three replicate ClickSeq libraries were generated from the same RNA sample to validate the reproducibility of ClickSeq and to determine the cut-off for sensitivity of discovery. Each point represents an individual recombination event and the x- and y- axes is the number of reads mapping to that specific event for each data set. The size of the point indicates the number of different events that share the same coordinates, as indicated by the key. These data illustrate the reproducibility with which recombination events are found when multiple libraries are generated side-by-side. Pearson correlation coefficients exceed 0.99 when comparing RNA1 or RNA2 recombination between each pair of replicates.
(PDF)

S3 Fig Conservation maps, similar to those illustrated in Fig 3 are shown for every passage in every replicate including the original inoculum (P0). (PDF)

S4 Fig Alignment of MinION reads. A composite snapshot of the TABLET sequence viewer alignment of RNA2 from the reads generated by the MinION (P4R2).
(PDF)

S5 Fig Error rate of the Oxford Nanopore MinION. **(A)** A histogram of the frequency of error rates over every mapped position across all Nanopore datasets is shown. Errors include substitutions, insertions and deletions shorter than 25 nts. The proportion of correct base matches to mismatches is shown on the x-axis. The y-axis indicates the number of nucleotide coordinates with the corresponding error rate. This reveals a mode and mean error rate of 5.0% and 6.3% respectively. Frequency of **(B)** deletions and **(C)** insertions within the MinION dataset.
(PDF)

S6 Fig **Stacked-area plot of showing the pathways of FHV DI-RNA evolution** for **(A)** RNA1 and **(B)** RNA2. Similar to **Fig 6**, except all species are included, including genomes represented by only one MinION nanopore read. The passage number is indicated on the x-axis and the stacked frequencies of each detected defective RNA is shown in the y-axis. Each non-contiguous color represents a specific genome characterized by MinION nanopore sequencing. Wild-type genomes are colored green, genomes with one deletion are colored in shades of blue, and genomes with two or more deletions are colored in shades of oranges (using the same color scheme as in **Fig 5C**).
(PDF)

S7 Fig Scatterplots showing live cell gating. Screenshots of the InCyte software (of the Guava easyCyte HT flow cytometer) indicating gating used to count live cells. **(A)** Uninfected S2 cells. **(B)** S2 cells four days post-infection with FHV exhibiting cytopathic effect.
(PDF)

S1 Table Full breakdown of ClickSeq read mapping results. Each passage in each replicate as well as the inoculum is shown, similar to **Table 1**.
(PDF)

S1 Datafile Raw virus recombination events data from *ViReMa* analysis of ClickSeq data. Each passage in each replicate as well as the inoculum is shown. Output format, as described in Routh et al. [32], is given as “*DonorCoord_to_AcceptorCoord_#_Counts”*. This is the raw data used to populate **Table 1**and **S1 Table**.
(ZIP)

S2 Datafile Genomes characterized by MinION nanopore sequencing. Table reports the annotated genomes (wild-type or defective) and the number of mapped reads in each passage. This the raw data used to populate the stacked-area plots in **Fig 6**and **S6 Fig**.
(XLSX)
