# Supplementary figures and images for: Parallel ClickSeq and Nanopore sequencing elucidates the rapid evolution of defective-interfering RNAs in Flock House virus
Source: PLoS Pathog. 2017 May 5;13(5):e1006365. doi: 10.1371/journal.ppat.1006365 (PMC5435362; doi:10.1371/journal.ppat.1006365)

Supplemental Figure 1:

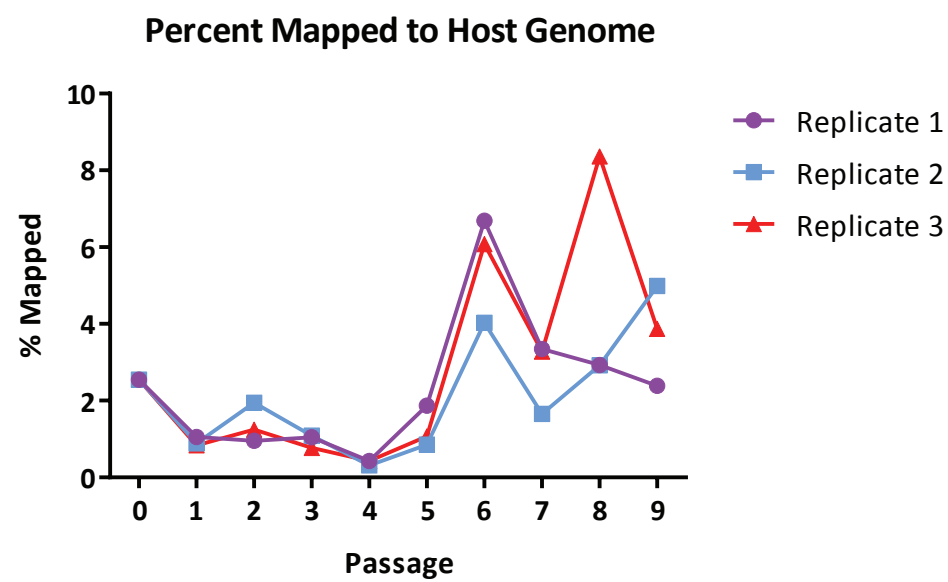

Supplement: S1 Fig — Percent mapping is calculated by the frequency of reads that mapped to the host genome compared to all processed reads. (PDF) [file ppat.1006365.s001.pdf]

Supplemental Figure 2:

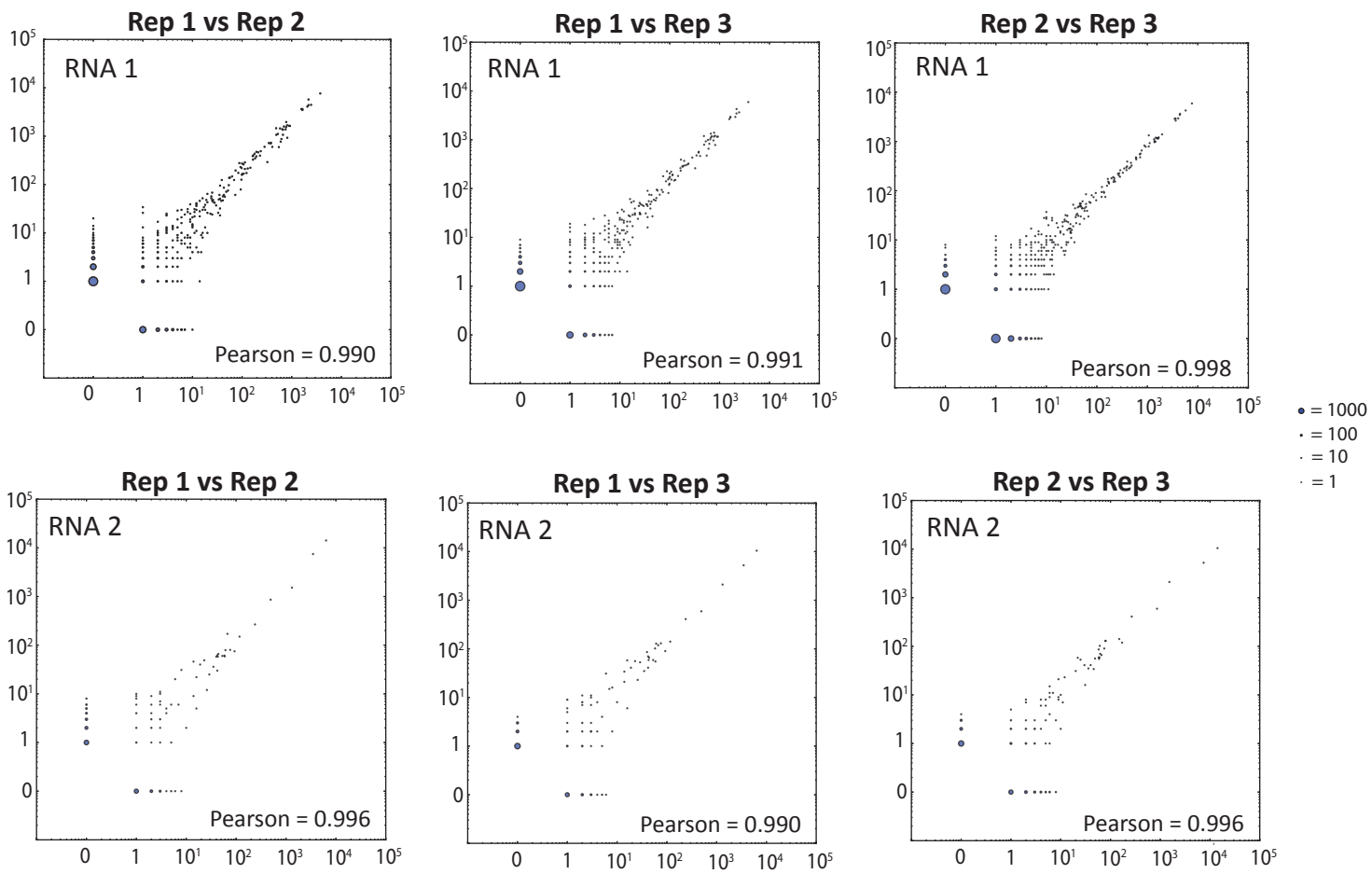

Supplement: S2 Fig — Three replicate ClickSeq libraries were generated from the same RNA sample to validate the reproducibility of ClickSeq and to determine the cut-off for sensitivity of discovery. Each point represents an individual recombination event and the x- and y- axes is the number of reads mapping to that specific event for each data set. The size of the point indicates the number of different events that share the same coordinates, as indicated by the key. These data illustrate the reproducibility with which recombination events are found when multiple libraries are generated side-by-side. Pearson correlation coefficients exceed 0.99 when comparing RNA1 or RNA2 recombination between each pair of replicates. (PDF) [file ppat.1006365.s002.pdf]

Supplemental Figure 3:

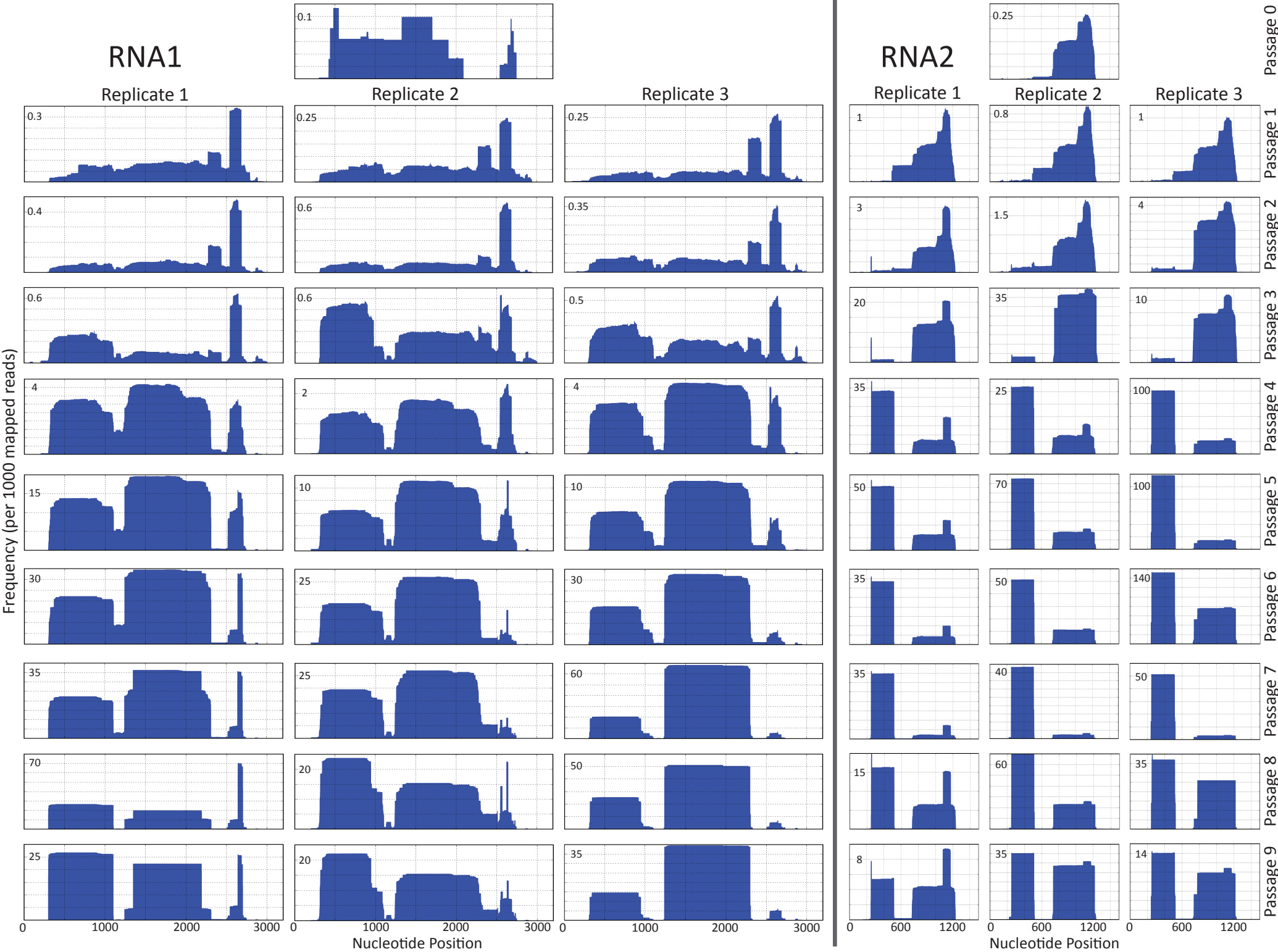

Supplement: S3 Fig — (PDF) [file ppat.1006365.s003.pdf]

Supplemental Figure 5:

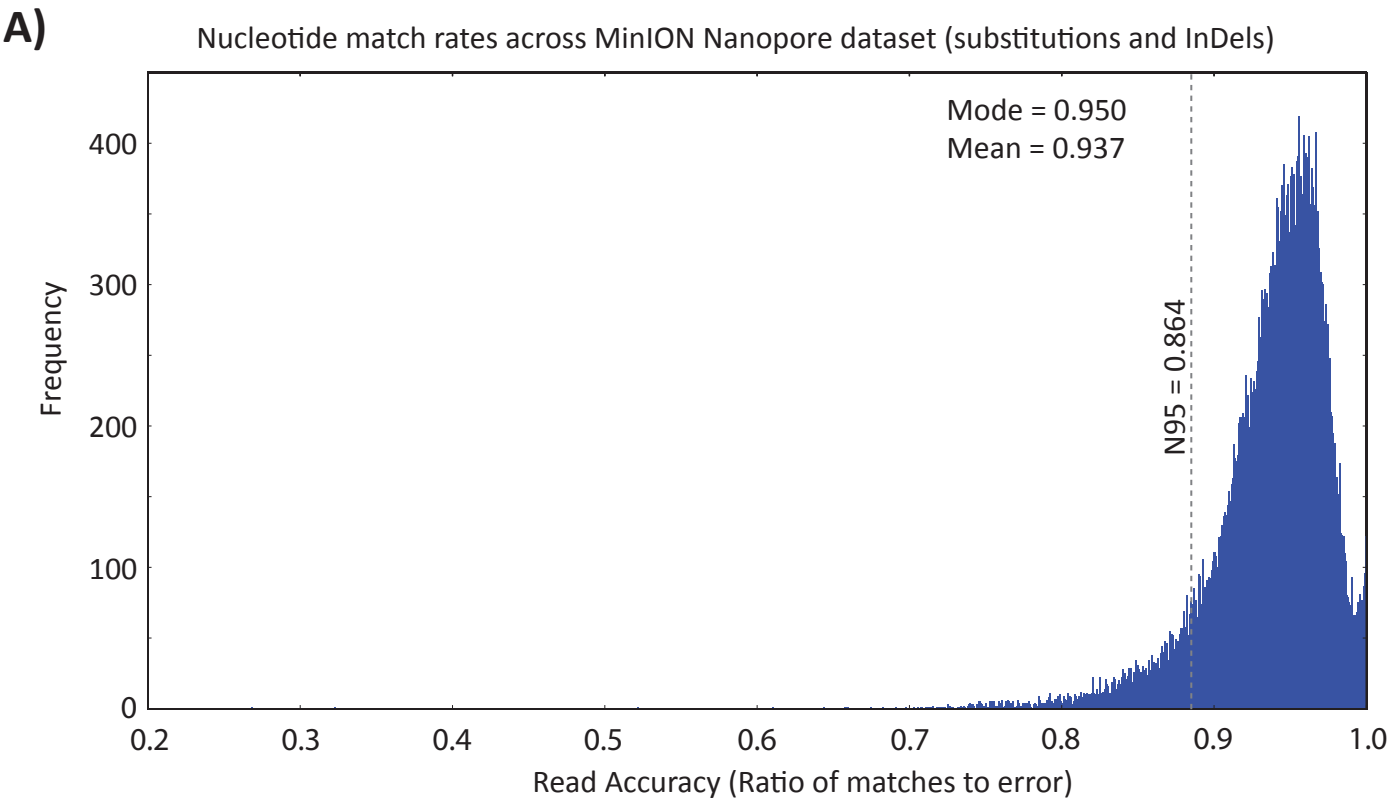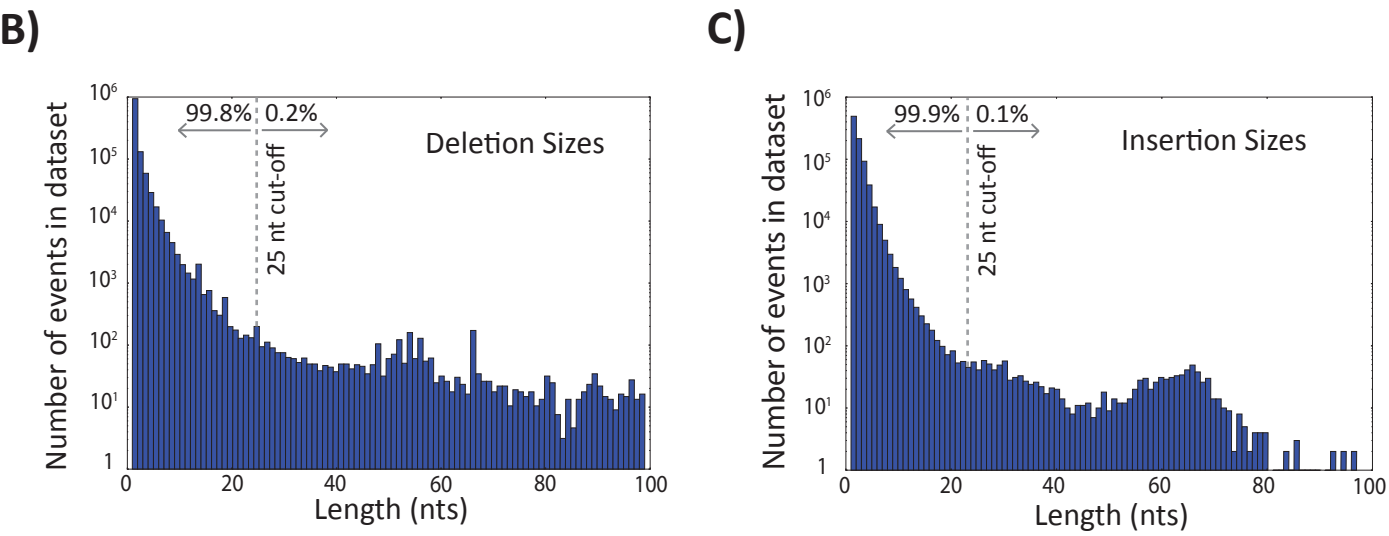

Supplement: S5 Fig — (A) A histogram of the frequency of error rates over every mapped position across all Nanopore datasets is shown. Errors include substitutions, insertions and deletions shorter than 25 nts. The proportion of correct base matches to mismatches is shown on the x-axis. The y-axis indicates the number of nucleotide coordinates with the corresponding error rate. This reveals a mode and mean error rate of 5.0% and 6.3% respectively. Frequency of (B) deletions and (C) insertions within the MinION dataset. (PDF) [file ppat.1006365.s005.pdf]

Supplemental Figure 6:

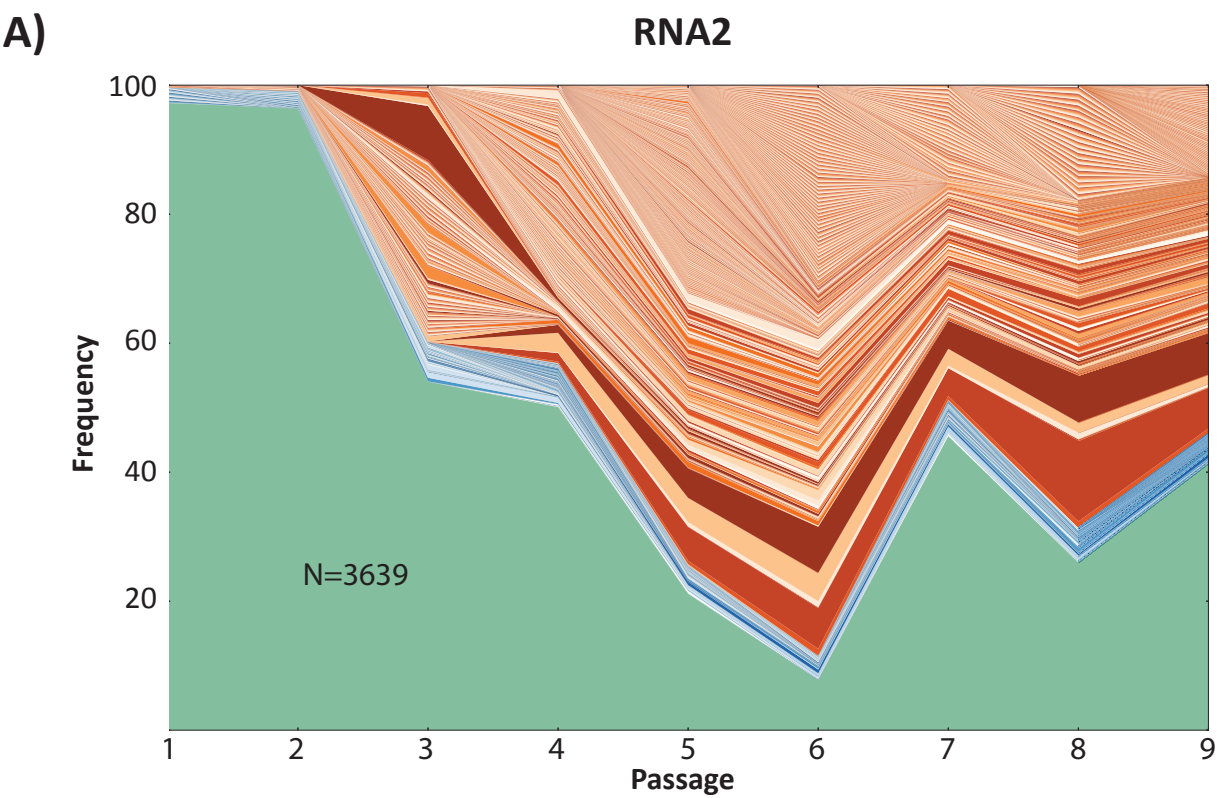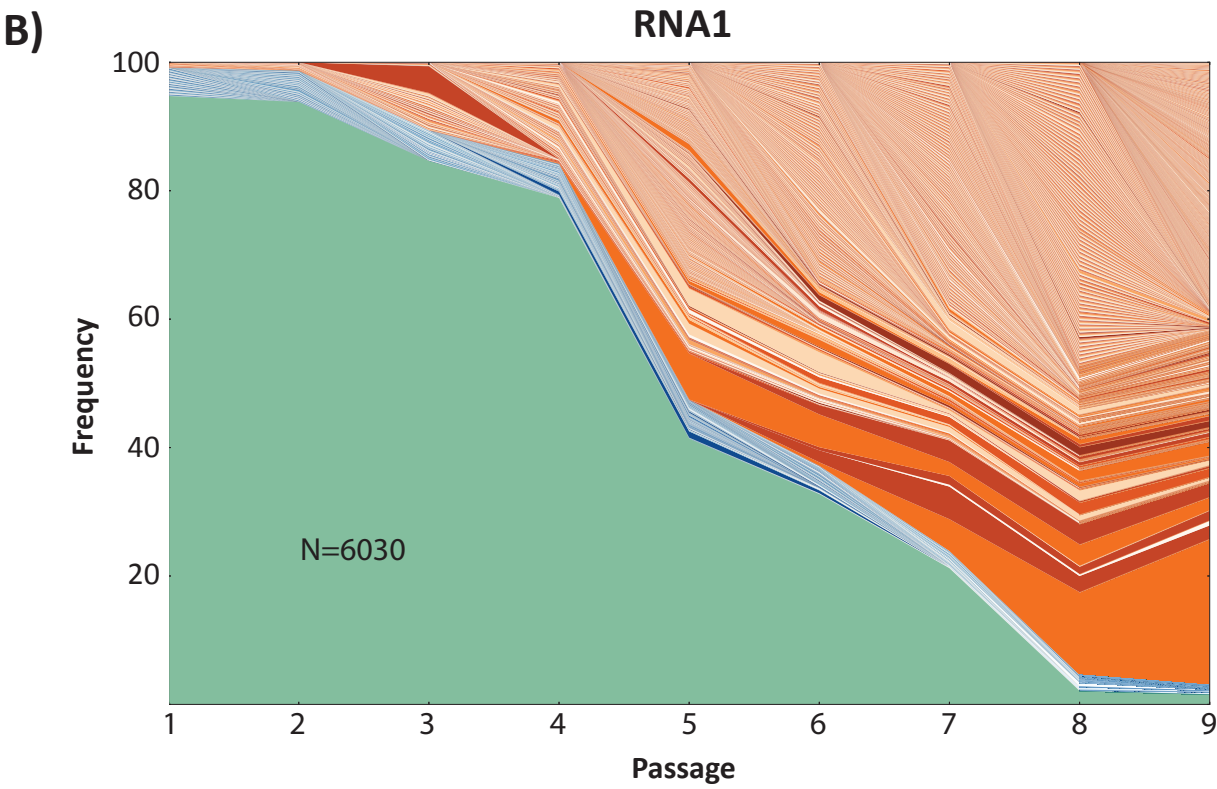

Supplement: S6 Fig — Stacked-area plot of showing the pathways of FHV DI-RNA evolution for (A) RNA1 and (B) RNA2. Similar to Fig 6, except all species are included, including genomes represented by only one MinION nanopore read. The passage number is indicated on the x-axis and the stacked frequencies of each detected defective RNA is shown in the y-axis. Each non-contiguous color represents a specific genome characterized by MinION nanopore sequencing. Wild-type genomes are colored green, genomes with one deletion are colored in shades of blue, and genomes with two or more deletions are colored in shades of oranges (using the same color scheme as in Fig 5C). (PDF) [file ppat.1006365.s006.pdf]

Supplemental Figure 7:

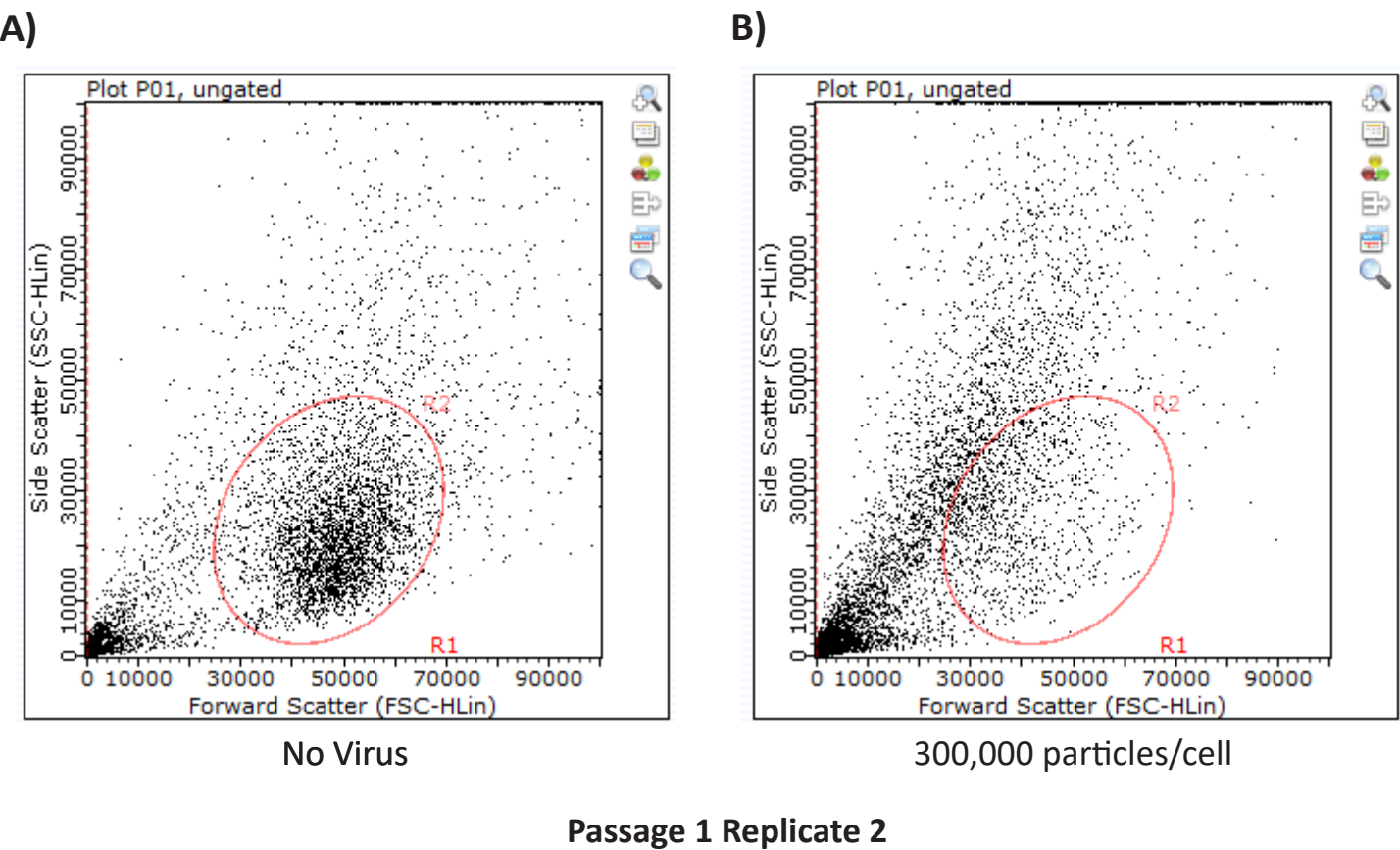

Supplement: S7 Fig — Screenshots of the InCyte software (of the Guava easyCyte HT flow cytometer) indicating gating used to count live cells. (A) Uninfected S2 cells. (B) S2 cells four days post-infection with FHV exhibiting cytopathic effect. (PDF) [file ppat.1006365.s007.pdf]
