# Supplementary material for: Parallel ClickSeq and Nanopore sequencing elucidates the rapid evolution of defective-interfering RNAs in Flock House virus
Source: PLoS Pathog. 2017 May 5;13(5):e1006365. doi: 10.1371/journal.ppat.1006365 (PMC5435362; doi:10.1371/journal.ppat.1006365)

Supplemental Figure 4:

Composit of TABLET Viewer alignment of Passage 4 MinION Nanopore data to FHV RNA 2

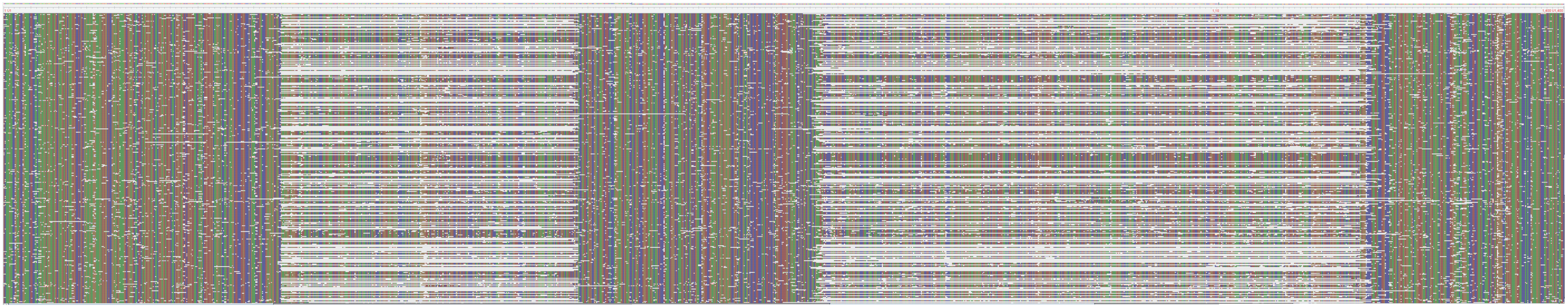

Supplement: S4 Fig — A composite snapshot of the TABLET sequence viewer alignment of RNA2 from the reads generated by the MinION (P4R2). (PDF) [file ppat.1006365.s004.pdf]
