# Supplementary material for: Parallel ClickSeq and Nanopore sequencing elucidates the rapid evolution of defective-interfering RNAs in Flock House virus
Source: PLoS Pathog. 2017 May 5;13(5):e1006365. doi: 10.1371/journal.ppat.1006365 (PMC5435362; doi:10.1371/journal.ppat.1006365)
Supplement: S1 Table — Each passage in each replicate as well as the inoculum is shown, similar to Table 1. (PDF) [file ppat.1006365.s008.pdf]

Supplemental Table 1:

Replicate 1	Passage 0	Passage 1	Passage 2	Passage 3	Passage 4	Passage 5	Passage 6	Passage 7	Passage 8	Passage 9
Total Reads	41 578 802	8 425 984	18 582 914	3 030 368	6 558 293	7 333 790	7 529 709	4 919 929	8 017 787	9 084 665
FHV Genome	39 595 900	8 271 467	18 241 065	2 954 304	6 365 376	6 720 739	6 309 807	4 293 229	6 830 523	8 145 020
D. melanogaster	1 057 037	89 123	177 811	31 802	28 179	137 249	503 422	164 721	234 721	216 618
FHV Recombinations	161 205	7 578	34 573	24 517	123 637	356 277	500 760	372 253	810 863	587 347
RNA1-RNA1	2 902	3 903	13 586	3 384	55 454	269 016	444 624	330 290	788 324	569 366
RNA2-RNA2	81 765	3 558	20 603	21 097	67 686	86 553	56 085	41 575	21 602	17 236
Inter-RNA	76 538	117	384	36	497	708	51	388	937	745
Other	141 332	5 730	11 198	2 807	8 639	26 571	38 128	19 573	39 776	33 892
Unmapped	139 057	3 451	4 501	1 441	2 167	2 713	8 805	2 184	2 686	3 257

Replicate 2	Passage 1	Passage 2	Passage 3	Passage 4	Passage 5	Passage 6	Passage 7	Passage 8	Passage 9
Total Reads	11 303 746	30 630 462	15 720 784	9 438 862	9 526 140	7 118 697	4 316 239	9 249 953	8 108 519
FHV Genome	11 121 111	29 693 227	15 243 341	9 229 809	8 996 228	6 293 323	3 953 100	8 162 144	7 088 432
D. melanogaster	100 716	596 462	171 518	29 353	81 172	286 605	71 238	270 156	404 742
FHV Recombinations	9 259	46 962	192 737	126 061	377 703	409 316	246 959	679 389	437 566
RNA1-RNA1	5 103	25 052	24 809	46 225	241 048	333 125	204 609	566 092	376 893
RNA2-RNA2	3 845	20 919	167 464	79 550	136 141	75 424	42 076	111 895	59 650
Inter-RNA	311	991	464	286	514	767	274	1 402	1 023
Other	7 358	33 488	16 642	8 934	18 569	33 152	16 553	46 621	55 529
Unmapped	3 779	9 879	3 026	2 952	3 569	4 084	1 631	2 690	3 549

Replicate 3	Passage 1	Passage 2	Passage 3	Passage 4	Passage 5	Passage 6	Passage 7	Passage 8	Passage 9
Total Reads	16 420 686	17 358 462	15 283 445	7 908 511	6 784 205	6 438 793	9 369 727	1 189 217	9 080 091
FHV Genome	16 161 893	12 543 802	14 995 997	7 561 609	6 388 461	5 340 577	8 044 482	946 913	7 892 118
D. melanogaster	137 322	215 958	117 684	33 721	72 896	391 630	307 068	99 476	351 639
FHV Recombinations	13 847	25 826	70 443	238 482	244 913	507 397	767 795	82 977	485 925
RNA1-RNA1	8 022	8 212	19 213	78 822	133 208	266 278	716 381	77 638	475 383
RNA2-RNA2	5 517	17 288	50 877	159 107	111 256	240 588	50 729	5 332	10 123
Inter-RNA	308	326	353	553	449	531	685	7	419
Other	9 893	31 671	10 589	36 275	41 337	79 144	149 528	31 665	235 665
Unmapped	6 467	205 049	6 456	2 613	3 211	3 043	2 903	1 227	2 928
